# Capturing vocal communication in a free-living corvid: high-resolution data from low-impact miniaturized tags

**DOI:** 10.1007/s10071-025-02018-0

**Published:** 2025-10-29

**Authors:** Vittorio Baglione, Daniela Canestrari, Maddie Cusimano, Benjamin Hoffman, Victor Moreno, Eva Trapote

**Affiliations:** 1 Departamento de Biodiversidad y Gestión Ambiental, Universidad de León, Campus de Vegazana s/n, 24071 León, Spain; 2 Earth Species Project, Berkeley, CA USA; 3 Departamento de Ciencias Agroforestales, Universidad de Valladolid, Palencia, Spain

**Keywords:** Vocal communication, Animal-born microphone, Corvid, Carrion crow

## Abstract

Understanding vocal communication is essential to unraveling avian social behavior and cognition; however, audio recording remains particularly challenging in field studies involving wild populations. In this study, we deployed a lightweight, multi-sensor biologging device (MiniDTAG) designed for medium- to large-sized birds. The device integrates a microphone, accelerometer, magnetometer, and pressure sensors into a 12.5 g package, enabling high-fidelity acoustic and behavioral data collection. We deployed 52 MiniDTAGs over three breeding seasons in free-ranging cooperatively breeding carrion crows (*Corvus corone*) in northern Spain. The auto-releasing attachment method allowed the birds to free themselves from the tag after 18.5 days, on average. We recovered 87% tags, collecting over 83 h of data per device on average. Using a machine learning model (Voxaboxen), we detected over 127,000 vocalizations and assigned them to focal tagged individuals, adult conspecifics, crow chicks, and parasitic great spotted cuckoo nestlings (*Clamator glandarius*) with high precision and recall. We also explored the potential of accelerometer data to identify specific behaviors within a cooperative context, namely anti-predator mobbing. To evaluate logger impact, we analyzed 825 h of video from 22 crow groups and found minimal effects on brood feeding rates and reproductive success. Our results highlight the MiniDTAG’s potential to advance the study of animal communication by capturing vocalizations across the whole range of amplitudes. This approach opens new avenues for exploring the mechanisms of cooperation and information exchange in complex social systems and lays the groundwork for future comparative studies in corvid communication.

**Supplementary Information:**

The online version contains supplementary material available at 10.1007/s10071-025-02018-0.

## Introduction

Communication provides a window into an animal’s inner states. For example, begging calls in bird nestlings inform parents about their hunger level (Kilner 2002), while courtship displays indicate an individual’s readiness to mate (e.g. Clotfelter et al. 2006). Importantly, communication both relies on and reflects underlying cognitive abilities. Examining communicative behaviour can shed light on animals’ mental representation of objects or organisms (e.g. predators; Seyfarth et al. 1980), their ability to navigate complex social structures and third parties’ social relationships (Slocombe and Zuberbühler 2007), their memory capacities (Bolhuis et al. 2010) and even their intentions to deceive or manipulate others (Wheeler 2009).

Vocal communication is paramount in birds, which are often the model species of bioacoustics studies (Stowell 2022). The first step toward decoding a species acoustic communication system is describing its vocal repertoire, a task that poses significant challenges, especially in field studies. Traditional recording methods that rely on directional microphones often skew a species’ vocal repertoire toward medium- to long-distance calls or songs, while overlooking many vocalizations used in close-range communication. In this respect, animal bio-logging - i.e. the use of small, animal-mounted electronic devices to record data - represents a valuable alternative, though its potential has not yet been fully explored. Animal-borne bio-loggers are becoming increasingly common to study animal biology (Wilmers et al. 2015). Bio-loggers collect a large variety of data (such as GPS location, environmental conditions, physiological and motion parameters, sounds, etc.) that allow addressing questions about physiology, migration, behaviour and social interactions in unprecedented detail (Wilmers et al. 2015). During the last 20 years, animal-borne bio-loggers have been miniaturized, their lifespan has been extended and their memory capacity improved (Evans et al. 2013), allowing their use on a larger variety of organisms. In addition, these advances now allow coupling different sensors into single devices to generate multi-channels data that have the potential to expand the range of biological and ecological questions that we can address (Wilmers et al. 2015).

Animal-borne microphones, in particular, provide an excellent opportunity to record animal and environmental sounds (Lynch et al. 2013), reducing human disturbance in free living animals. They also enable the recording of the quietest sounds, such as short-range vocalizations (Lynch et al. 2013), as well as calls emitted in locations outside the reach of classical directed microphones (for example, Cape Gannets *Morus capensis* at sea; see Thiebault et al. 2016). These devices have, therefore, the potential of sampling the complete repertoire of individuals, generating data on their vocal activity in unprecedented detail. In addition, the background sound that animal-borne microphones register can help to infer behaviours, like diving or landing in water in Cape gannets (Thiebault et al. 2021), hunting events of Canada Lynx *Lynx canadiensis* (Studd et al. 2021) and even to recognize social contexts in Jackdaws *Corvus monedula* as well as heterospecific callers (Stowell et al. 2017).

Decoding animal communication systems greatly benefits from matching individual vocalizations with the behaviour of the caller and the environmental/social context where they were emitted (Rutz et al. 2023). The combination of microphones with other sensors might provide this key information. Tri-axial accelerometers and magnetometers can be used to infer animal posture and behaviour, and to estimate energy expenditure (Wilson et al. 2008, 2020). They have been widely used to study animal behaviour in a variety of terrestrial, aquatic and flying animals (Shepard et al. 2008; Shamoun-Baranes et al. 2012; Wilson et al. 2008; Williams et al. 2017; Conners et al. 2021).

The exciting new opportunities that biologging offers to bioacoustics research, however, are not without challenges. It has been shown that bio-loggers can have detrimental impacts, ranging from a reduction of movement, increase of comfort behaviours and even physical damage (Thaxter et al. 2016), and that these effects can translate into reduced reproductive success and survival. Flying animals may be especially affected. Barron et al. (2010) reported on behavioural modifications, increased energy expenditure or reduced breeding success in birds carrying devices. Perhaps surprisingly, they found no evidence that heavier devices had proportionally greater effects and put into question the “5% body mass rule” (3% for some researchers; Kenward 2001) that generalizes a “safety threshold” for all flying animals. In fact, the placement of the tag may be more important than its weight (Vandenabeele et al. 2012, 2014; Bowlin et al. 2010). Therefore, although reducing tag weight is always advisable, the effects of each device should be carefully assessed on a case-by-case basis. The method of attaching a device to an animal’s body can significantly impact its welfare (Bodey et al. 2017) and, in the case of acoustic recorders, may also influence the quality of the recorded sounds. Additionally, the necessity of recapturing tagged individuals to retrieve the logger can induce further stress, potentially leading to long-term consequences for the animals.

Despite their potential advantages, animal-borne multi-sensor devices have rarely been used to study acoustic communication in free-living birds, particularly in medium- and small-sized species, where optimizing the trade-off between device weight, sensor combination, and storage capacity presents a significant challenge. A further difficulty lies in the need to develop and implement automated data processing tools, as detecting target vocalizations within the extensive audio recordings generated by on-bird microphones is virtually impossible to manage manually (Marchal et al. 2021).

We deployed a miniaturized, fully programmable, multichannel data logger (MiniDTAG) on a medium-sized corvid species, the carrion crow. Corvids are key model species for investigating acoustic communication in animals (Wascher and Reynolds 2025). Their advanced cognitive abilities (Emery and Clayton 2004), coupled with substantial inter- and intraspecific variability in social structures—ranging from socially territorial monogamy to colonial and cooperative group living—offer a unique opportunity to explore the role of communication in navigating complex social environments. Carrion crows, in particular, form complex social units consisting of stable kin groups that include a breeding pair, non-dispersing offspring, and more distantly related immigrants (Baglione et al. 2003). These groups engage in cooperative behaviors such as joint brood care, territorial defense, and predator deterrence. Theoretical work suggests that cooperation depends on the degree of coordination between participants, which in turn is critically influenced by the amount of information they exchange (Nöe 2006, Miglino et al. 2008). Close-range acoustic calls among group members are therefore likely to play a key role in enabling the complex forms of cooperation observed in carrion crows. Animal-borne microphones present a promising method for capturing these calls; however, there is currently little to no data on their feasibility, advantages, and potential limitations when deployed in the wild on a medium-sized, highly neophobic, and cognitively complex bird species.

In this study, we described and tested the MiniDTAG and its attachment/detachment method with the following objectives: (1) to report the amount of data collected, the types and quality of vocalizations recorded, the tag failure rate and the tag retrieval rate; (2) to develop and evaluate a machine learning detection model capable of distinguishing between the calls of focal adults (i.e., individuals carrying the tag), non-focal adults, crow nestlings and great spotted cuckoo (*Clamator glandarius*) nestlings (a common brood parasite of crows; Baglione et al. 2017) in long biologger recordings; (3) to assess the potential of integrating information from the accelerometer to identify specific behaviours within a cooperative context, namely predator deterrence (4) to evaluate the impact of the MiniDTAG on crow brood provisioning behaviour and reproductive success.

## Methods

### MiniDTAG design

**Fig. 1:**
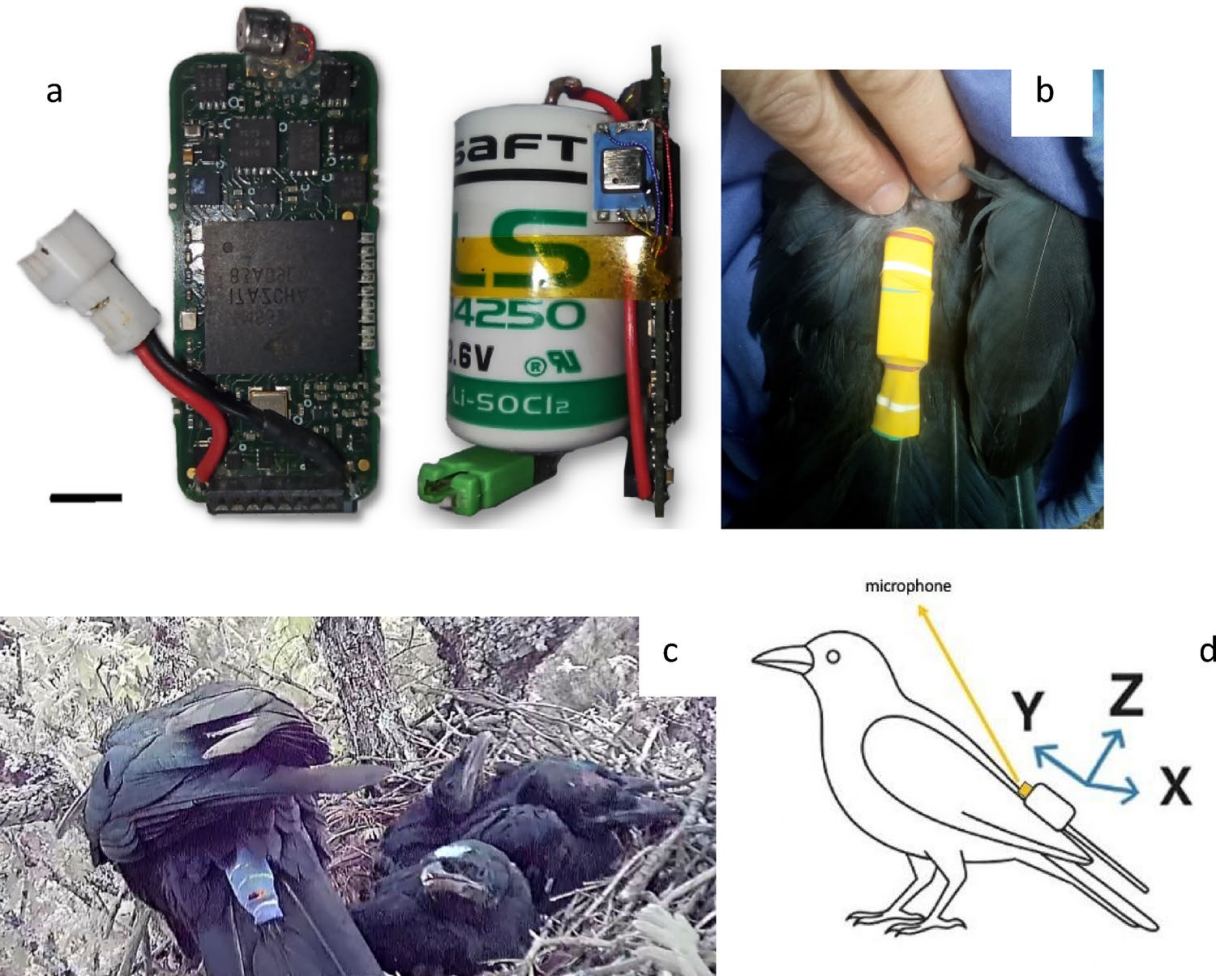
(**a**) Detail of miniDTAG (left); the tag with battery (right); scale line is 5 mm. (**b**) MiniDTAG deployed on the tail of a carrion crow; (**c**) tagged crow at the nest. (**d**) Schematic showing biologger arrangement with accelerometer axes. Positive x-axis: rightward, y-axis: forward, z-axis: upward

The miniDTAG was adapted from a 2.6-g bat tag integrating microphone, tri-axial accelerometer and tri-axial magnetometer (Stidsholt et al. 2019) with changes that enable long duration recordings on medium-sized birds. The tag (Fig. 1a) comprises a single custom-designed printed circuit board (PCB), onto which a 1.2 Ah lithium primary battery (Saft LS14250) is mounted. The 0.5 mm thick fibreglass (FR4) PCB houses a microphone preamplifier, anti-alias filter, 16-bit analog-to-digital converter, tri-axial accelerometer, tri-axial magnetometer, pressure sensor, temperature sensor and a 32 GB flash memory card. A low power digital signal processor on the board controls sampling of the sensors and performs lossless compression and error-correction on the data streams before saving them to the flash memory. After battery mounting, all components are protected with a heat shrink tube. The overall package measures 17 × 30 × 14 mm and weighs 12.5 g. Its size is well suited for a bird like the carrion crow, whose total body length and body mass range respectively between 480 and 560 mm and 396–602 g (Madge and Burn 1999). The current consumption of the tag when sampling all sensors is 6–7 mA and the battery supports continuous recording for about 6 days in laboratory settings (stable room temperature − 20 to 25 °C- fully charged battery, and tag kept stationary). The microphone signal is sampled at 93.75 kHz and is decimated by two (i.e., to a sampling rate of 46.875 kHz) before storing to memory. Analog and digital anti-alias filters set the upper frequency limit of the sound recording to 20 kHz. A single-pole high pass filter with a −3 dB frequency of 500 Hz is used to reduce the incidence of overloading due to wind noise during flying. The tri-axial accelerometer (Kionix KX022-1020 configured for ± 8 g full scale, 16-bit resolution) is sampled at 1000 Hz and decimated to a sampling rate of 200 Hz before saving to flash memory. The tri-axial magnetometer (ST Inc. LIS3MDL configured for ± 4 gauss full scale) is sampled at 50 Hz without anti-alias filter as the sensor is turned off between samples. The lack of an anti-alias filter is acceptable because of the lower frequency content of magnetometer (i.e., orientation) data compared to acceleration (Martín Lopez et al. 2016). The sensor and audio sampling rates are derived from a common clock and so maintain timing synchrony throughout the recording.

### Study species and device attachment

Carrion crows are medium size passerines broadly distributed in Europe and Asia. Since 1995, we have been carrying out a long-term study on a population in a rural area of northern Spain (42º 37’N, 51 26’ W), where crows breed cooperatively in kin groups of 3–9 members (mean ± SE = 3.2 ± 0.08) that defend exclusive, all-purpose territories (Baglione et al. 2002).

Between March and June each year, breeding territories were surveyed to locate nests and to monitor crow reproduction. In 2018, 2019 and 2021 we selected 10–15 territories per year to deploy the miniDTAG on adult crows and to video record their brood provisioning behaviour when the chicks were 10–15 days old. We used camouflaged, miniaturized cameras mounted 50–100 cm from the nest on a suitable tree branch, positioned to provide a complete view of the nest and visiting birds. A digital video recorder placed at the base of the tree, powered by a 12 V car battery, completed the recording system (see Canestrari et al. 2005 for further details on the video setup). Adult carrion crows were trapped using two-compartment walk-in traps or an 8 ft. diameter snap trap (Northwoods Falconry) and banded with colour rings and wing tags following the protocols described in Baglione et al. (2002). All birds were measured, and 50–200 µl of blood were taken for molecular sexing (see Canestrari et al. 2023 for details).

We deployed 52 miniDTAGs, each packaged with a 0.6 g micro radio transmitter (Biotrack PicoPip). Birds were also fitted with wing tags and a coloured ring for distant visual identification and ultimately carried a total load of 19 g. The miniDTAG was attached to the two central tail feathers with a piece of the stem of a coloured balloon (Fig. 1b and c) following the procedure described in Rutz and Troscianko (2013). Briefly, the packaged logger was inserted into a tube of balloon that was held open by metal tongs. The two central tail feathers were then introduced into the balloon tube with the aid of the tongs. Once the logger was positioned at the base of the tail, with the microphone oriented towards the bird’s head (Fig. 1d), the metal tongs were slowly removed and the balloon contracted, securing the logger to the feathers without using any glue. A small piece of polystyrene, glued centrally at the bottom side of the logger, maintained the two tail feathers separated and prevented crossing-over. The VHF antenna was attached to the shaft of a tail feather with a drop of Loctite glue to avoid noisy movements. The thin rubber balloon material progressively deteriorated and finally broke, letting the miniDTAG fall to the ground, where it was radio-tracked using a Biotrack Sika receiver. Therefore, the placement of the miniDTAG on the tail feathers (e.g., as opposed to back-mounted tags) and its attachment with rubber made recapture unnecessary.

### Sound data and detection of crow vocalizations

A deployment for one individual was chunked into time periods, with each time period having an associated accelerometer plus magnetometer file, audio file, and metadata files. Each file was marked with the internal clock time. Most often, audio files were nearly continuous in time (gap of less than 0.0001 s) but may also have overlapped for minutes or, in rare cases, had large gaps between them (> 20 h). Overall, 3.2% of file transitions were longer than one hour. We use the term “deployment data” to refer to all files for a single individual and “file” to refer to one chunk of the appropriate modality.

In order to detect and characterize vocal activity recorded by the tags, we developed a detection model using the detection framework Voxaboxen (Mahon et al. 2025). The motivation behind Voxaboxen was to use an object detection framework, first developed for computer vision tasks, in order to predict bounding boxes (i.e. start- and stop-times) of vocalizations in audio. The advantage of this approach is that, unlike frame-based approaches described in Cohen et al. (2020), an object detection framework allows the model to put a separate bounding box around each vocalization of interest, even when these vocalizations are overlapping. We refer the reader to Stowell (2022) for a more detailed description of the differences between these approaches.

To develop our detection model, we first annotated a subset of files with bounding boxes around each vocalization, and then used this dataset to train our detection model. We evaluated the trained model’s performance on annotated data that was held-out from training and used it to detect vocalizations in all the deployment data made by the tags. We now describe these steps in detail.

**Fig. 2:**
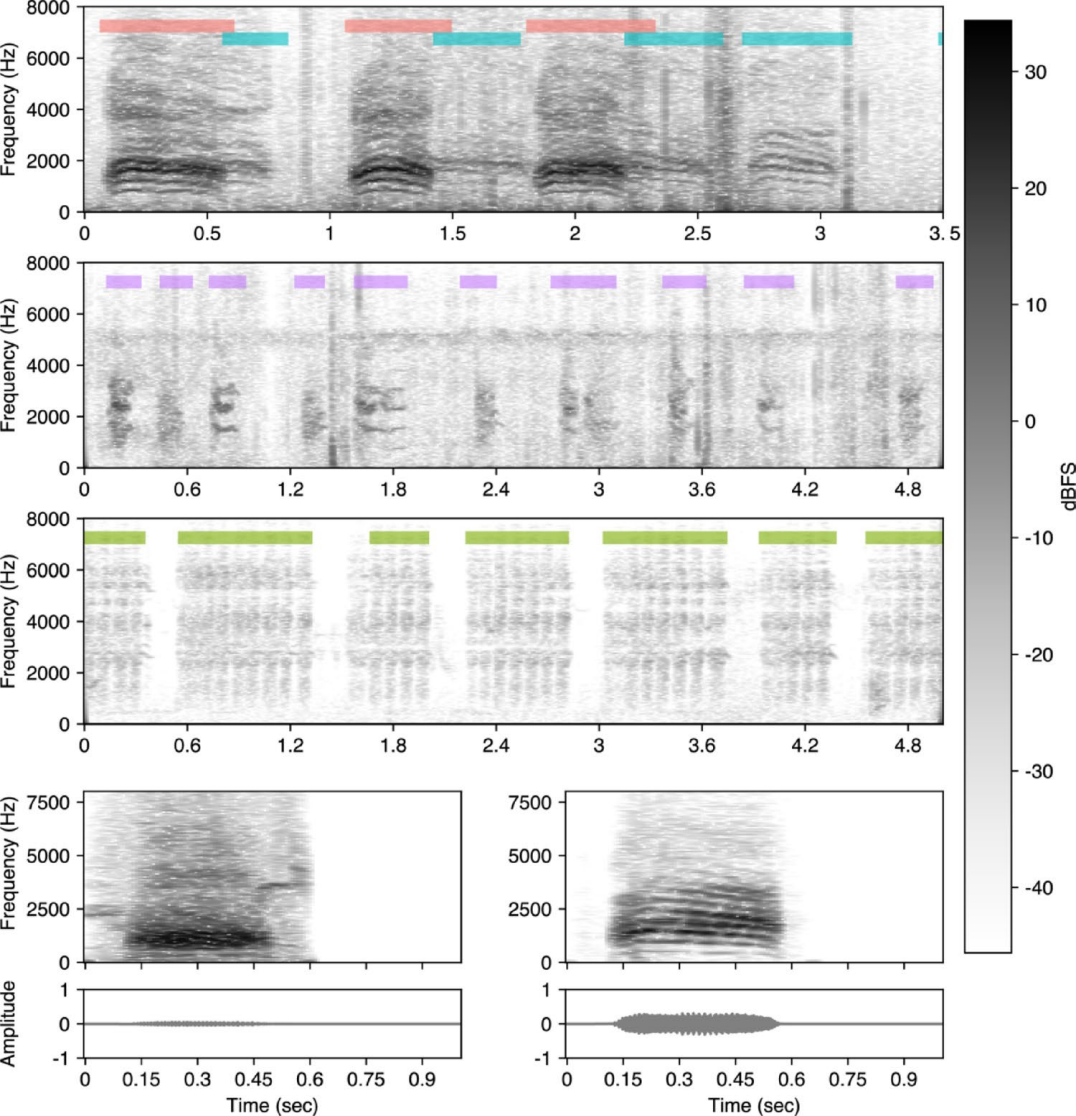
Spectrograms of examples from biologger audio. Top: vocalization detections classified as *Focal* (red) or *Non*-*focal* (blue). In this example, the non-focal vocalizations are quieter and have relatively less high-frequency energy than the focal vocalizations, both cues to the distance of the non-focal caller from the focal microphone. Second row: annotated crow chick vocalizations. Third row: annotated cuckoo chick vocalizations. The cuckoo vocalizations are relatively broadband and show repeating stereotyped segments. Bottom: spectrograms and waveforms of two different kinds of vocalizations, a relatively quiet (left) and loud (right) one

One of us (DC) annotated 35 audio files taken from tags on 14 birds, all recorded in 2019. The files ranged between 1.6 and 8.2 h and had a total duration of 187.7 h. Annotations were made in Raven Pro (The Cornell Lab of Ornithology 2019), where spectrograms were generated using default settings (Hann window; DFT length = 512, window length = 512, overlap = 50%). Crow vocalizations occur in discrete units separated by periods of silence. Each annotated bounding box included the start- and stop- time of the vocalization, which was typically characterized by an abrupt change in amplitude, as well as a category label. Annotations did not include frequency information. We used four categories (Fig. 2): *Focal*, if the vocalization was made by the tagged crow; *Non-focal*, if the vocalization was made by another adult crow; *Crow chick*, if the vocalization was made by a crow nestling; and *Cuckoo*, if the vocalization was made by a great spotted cuckoo nestling, the brood parasite that is often raised in crow nests in this population (Baglione et al. 2017). Additionally, crow vocalizations could be marked as *Unknown* if it was difficult to determine whether they were produced by focal or non-focal individuals (how the model deals with these annotations is described below). Cuckoo and crow chick vocalizations are typically begging calls and are readily distinguishable from adult vocalizations. Crow nestling begging calls are high-pitched, whining, and highly repetitive, and are typically produced in short bursts when parents are near. In contrast, great spotted cuckoo nestlings produce more persistent and intense begging calls that allow them to compete for food with the larger crow nest mates (Fig. 2, middle rows). Distinguishing between focal and non-focal vocalizations (Fig. 2, top) based solely on amplitude could be difficult in some cases, since it is possible for nearby non-focal calls to appear louder than focal ones due to differences in call strength and spatial arrangement. To improve accuracy, vocalizations were evaluated in context, using multiple cues such as environmental filtering, changes in intensity, and movement-related changes in sound. Crows’ tendency to repeat calls in bouts also aided identification. However, close proximity between birds can weaken these cues, leading to potential misclassifications, which we avoided through the use of the *Unknown* category (N. of *Focal* vocalisations = 4599, *Non-focal* = 1922, *Unknown* = 1988). Given these precautions, misassignments are unlikely to explain the observed prevalence of quiet vocalizations (see Results). Of the rest of annotated vocalizations, 14,447 were *Crow chick and* 14,587 were *Cuckoo*. Vocalizations had a median duration of 0.33 s. From the annotated files, we used 19 files from 7 individuals to train our detection model. The remaining 16 files came from 7 different individuals and were used to test model performance.

The detection model was based on an early version of Voxaboxen (Mahon et al. 2025). For the feature extractor, we used AVES-bio (Hagiwara 2023), which is a transformer-based architecture that was pre-trained on 360 h of animal audio. We did not use the bidirectional prediction option described in (Mahon et al. 2025). The model was trained to predict the start- and stop-times of calls, and assign each call to one of the four categories (*Focal*,* Non-focal*, *Crow chick*, and *Cuckoo*). During training, vocalizations annotated as *Unknown* were assigned to have equal probability across each of the four classes for the purpose of computing training loss; this meant that the model would learn to detect the vocalization but make the class label prediction with low confidence. During inference, in cases where the model had less than 50% confidence that the call belonged to one of these categories, the call was still detected but marked as *Unknown*.

To evaluate model performance, we matched each predicted bounding box with at most one annotated bounding box (and each annotated bounding box with at most one predicted bounding box). To do so, we followed the evaluation procedure described in Mahon et al. (2025 ), using an intersection-over-union (IoU) threshold of 0.5. This means that in order for a predicted box to be matched with an annotated bounding box, the area of their intersection divided by the area of their union must exceed 0.5. Once box predictions were matched with annotations, for each of the four categories, we computed classification precision, recall, and F1 scores. We also computed macro-averaged precision, recall, and F1 scores, by taking the average of these scores across the four categories. During evaluation, the model’s performance was not penalized if it failed to detect an *Unknown* vocalization.

To tune the learning rate hyperparameter, we did the following. From the 19 train files, we set aside 10% of each file (6 min per hour) as a validation set, which we used to compare different models trained with learning rates chosen from {1e-5, 5e-5, 1e-4}. We selected the learning rate (5e-5) that yielded the best macro F1 performance on the validation set and re-trained a model using this learning rate on the full train files. Other training hyperparameters (batch size = 4, clip duration = 6 s, n epochs = 25) were fixed, based on prior experience.

### Acoustic analyses of vocalization level and signal-to-noise ratio

We measured the level of focal vocalizations to assess the extent of production of low amplitude vocalizations. We compared this to the level of detected chick vocalizations. To obtain focal vocalizations, we retrieved all vocalizations with detection probability greater than 0.5 and *Focal* class probability greater than 0.5. This resulted in a dataset of 127,750 adult focal vocalizations that was used for measuring level and signal-to-noise ratio (see below). To obtain crow chick vocalizations, we first selected all detected crow chick vocalizations with detection probability > 0.5 and *Crow chick* class probability > 0.5. We then randomly sampled 100 crow chick vocalizations from each tagged individual, on each day where at least 100 crow chick vocalizations were detected. This resulted in a dataset of 18,900 crow chick vocalizations that was used for measuring level. For all vocalizations, we measured the level in decibels relative to full scale (dBFS).

To determine whether biologgers obtain sounds appropriate for further analyses of vocal repertoire of crow adults, we estimated the signal-to-noise ratio of the selected focal vocalizations. The focal vocalizations were considered as signal, while all other sources (including non-focal or chick vocalizations) were considered noise. To estimate the level of a focal vocalization, we measured the root-mean-square amplitude of the audio waveform from the onset to the offset of the detected vocalization. To measure the noise level, we retrieved four seconds of audio nearest to the focal vocalization, which did not contain another focal vocalization. We computed the root-mean-square amplitude of these four seconds. Then we estimated the signal-to-noise ratio as the ratio of these two amplitudes, in decibels. We did not apply any denoising algorithm which may improve the SNR (see Discussion). We filtered out any sounds with SNR > 60 dB (*N* = 15) as we found these contained clipping artifacts, and then measured the distribution of estimated SNR for each focal individual.

### Acceleration data

Accelerometer measurements were automatically calibrated using the d4findcal and do_cal functions from https://github.com/animaltags/tagtools_matlab, following guidance provided by the software’s author. The sensor channel was decimated by a factor of 4 before calibration, resulting in a sampling rate of 50 Hz. The tri-axial acceleration channel was normalized, so the average magnitude of the acceleration vector was equal to 1. The potential of accelerometer data to describe broad categories of bird behaviour (e.g., walking, flying, gliding, resting) is well established (Thiebault et al. 2016), and has also proven useful in the carrion crow, as demonstrated in a previous study (Hoffman et al. 2024; see Discussion). In this study, we aimed to further explore the potential of this sensor to infer behaviours particularly relevant for understanding the dynamics of crow cooperation and the role of vocal communication in shaping it.

We focused on anti-predator defence, which, alongside brood provisioning, is a key cooperative task undertaken by crow group members. To investigate this, we simulated an intrusion into the breeding territory using a staffed goshawk (*Accipiter gentilis*), the main avian predator of both adult crows and nestlings in the study population. The model was placed approximately 100 m from the nest, and the focal group’s response was observed for one hour from a concealed hide.

Control sessions involved the presentation of either a stuffed feral pigeon (*Columba livia*) or a black-headed gull (*Larus ridibundus*), with the order of control and experimental stimuli randomized across territories. Based on prior observations, crows typically respond to predators with loud vocalizations and, at times, by performing risky aerial dives, nearly striking the predator’s head or shoulders.

The simulated predator intrusions elicited strong defensive reactions from the groups, qualitatively similar to those observed during natural predation events. In contrast, control stimuli were invariably ignored. A hidden observer recorded all dives performed against the goshawk using a voice recorder. Two camouflaged video cameras at the experimental site aided in identifying participating individuals and quantifying the number of dives. The experiment was conducted in 17 territories, documenting the antipredator behaviour of 44 individuals, 5 of which carried a working miniDTAG.

To determine whether the acceleration data could characterize anti-predatory dives, we compared annotated dive segments with other flight segments not containing anti-predatory dives. We observed that the y- and z-direction of the static acceleration sometimes contained strong peaks, presumably due to banking forward in a dive, and hypothesized that this feature could distinguish flight types. To obtain dive segments, we extracted 2-minute clips centered on timepoints annotated as dives. These timepoints were identified by visual observation of the birds and were annotated as instantaneous events. If any clips overlapped, we combined them into one clip longer than 2-minutes. To obtain segments corresponding to dives, we split the two-minute clip into segments separated by relatively low amplitude (5 s of vector magnitude < 0.2 g) and kept any segments longer than 20 s. This resulted in 20 dive segments from four individuals.

To obtain non-dive segments, we extracted 20 segments of acceleration data occurring after nest visits. These segments corresponded to a flight away from the nest from takeoff to landing and were manually identified by finding the first flight sounds (e.g., wing flapping, wind, landing) that occurred after a period with detections of chick vocalizations. Flights away from the nest, as compared to other flights, are likely to be quite similar to dives since they are usually downward from the nest. Therefore, this comparison is likely more difficult than arbitrarily chosen flights. The flights were chosen to be at least ten seconds long and to occur as soon as possible before the experiment started.

We computed the static acceleration in the z-direction as follows. In each clip, we normalized the accelerometer channels such that the field strength equaled 1. We obtained the dynamic component of the acceleration by applying a high-pass delay free filter (cutoff = 3 Hz, chosen by visual inspection) using a linear phase symmetric FIR filter with a Hamming window, followed by group delay correction. The dynamic component was subtracted from the raw acceleration to obtain the static component. To find peaks, we used find_peaks in scipy 1.8.1 with parameters height = 1.8 and distance = 50 (corresponding to 1-second minimum between peaks). We then computed the proportion of segments that contained at least one peak, for each category.

For visualization, we also computed the spectrogram of the raw acceleration vector in the z-direction using stft and specshow of librosa version 0.10.0, with parameters n_fft = 128, hop_length = 12, win_length = 128.

### MiniDTAG impact

The impact of the miniDTAG was assessed by comparing the brood provisioning behaviour of tagged and non-tagged crows. Based on 825 h of nest video recording of 22 different groups, comprising 43 tagged and 14 untagged birds, we calculated the individual provisioning effort as the hourly frequency of feeds, defined as every event of food regurgitation into a chick open gape. Previous studies showed that this measure correlates with the actual amount of food brought to the nest, and it is therefore suitable to estimate parental care effort (Canestrari et al. 2005). We used a Linear Mixed Model fitted by Restricted Maximum Likelihood (REML) in R (R Core Team 2022), using deployment of the miniDTAG (yes/no), social category (breeder/helper), group size, brood size, Julian laying date and time of the day (morning, from 5:00 to 10:00; midday, from 10:01 to 15:00; afternoon, from 15:01 to 20:00; all times in CEST) as fixed terms, and individual identity as a random term. The response term was log-transformed to improve the normality of the residuals. Normality of DHARMa scaled residuals, heteroscedasticity and outliers were checked with the package *DHARMa* (Hartig 2022), while multicollinearity between fixed factors was tested by computing variance inflation factors (VIFs) with the package *performance* (Lüdecke 2021). Additionally, we investigated whether either (i) the presence/absence or (ii) the total number of tagged individuals in a reproductive group influenced the number of offspring produced. The two General Linear Models, fitted with a Poisson distribution and a log link function, included group size, clutch size and year as fixed terms, and were checked as explained above. The sample included 42 nests surveyed in 2018 and 2019.

## Results

### Loggers’ retrieval and data recording

**Fig. 3:**
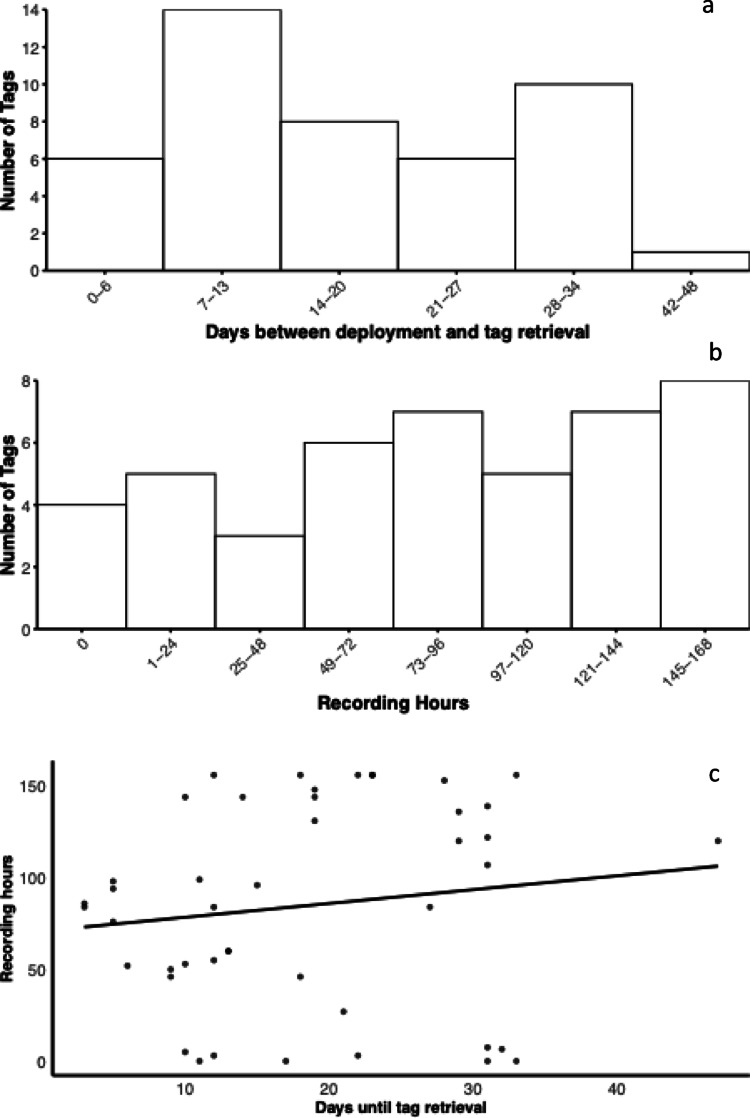
(**a**) Number of days elapsed between deployment and retrieval of the miniDTAGs; (**b**) Number of hours recorded by each tag; (**c**) Correlation between the duration of audio-files successfully downloaded from a tag and its exposure to outdoor conditions

Seven loggers could not be retrieved, while the rest were found within 47 days from deployment. The mean ± SE (Standard Errors are provided for all mean values hereafter) number of days between deployment and retrieval was 18.5 ± 1.41 days (range 3–47 days; Fig. 3a). All tags, except one, were recovered within the corresponding crow territories. In this regard, the territorial and sedentary behavior of the studied crows (Baglione et al. 2005) proved advantageous for locating the loggers. The tagged birds were radio-tracked frequently, although we could not follow a standardized schedule. A stationary signal indicated that the device had detached and was therefore searched intensively. Therefore, although the exact permanence of the logger on each bird could not be exactly measured, it can roughly approximate the time between depletion and retrieval (reported above) in most cases. Five tags detached before battery depletion. The number of recorded hours per tag also varied (mean = 83.15 ± 8.25 h; range 0–156 h; Fig. 3b), with 66% of the loggers not completing their 6 days expected cycle. The number of hours recorded and successfully downloaded did not vary depending on the number of days elapsed between deployment and retrieval (Spearman *R* = 0.18, *p* = 0.24, *N* = 45; Fig. 3c).

### Detection of crow vocalizations

**Fig. 4:**
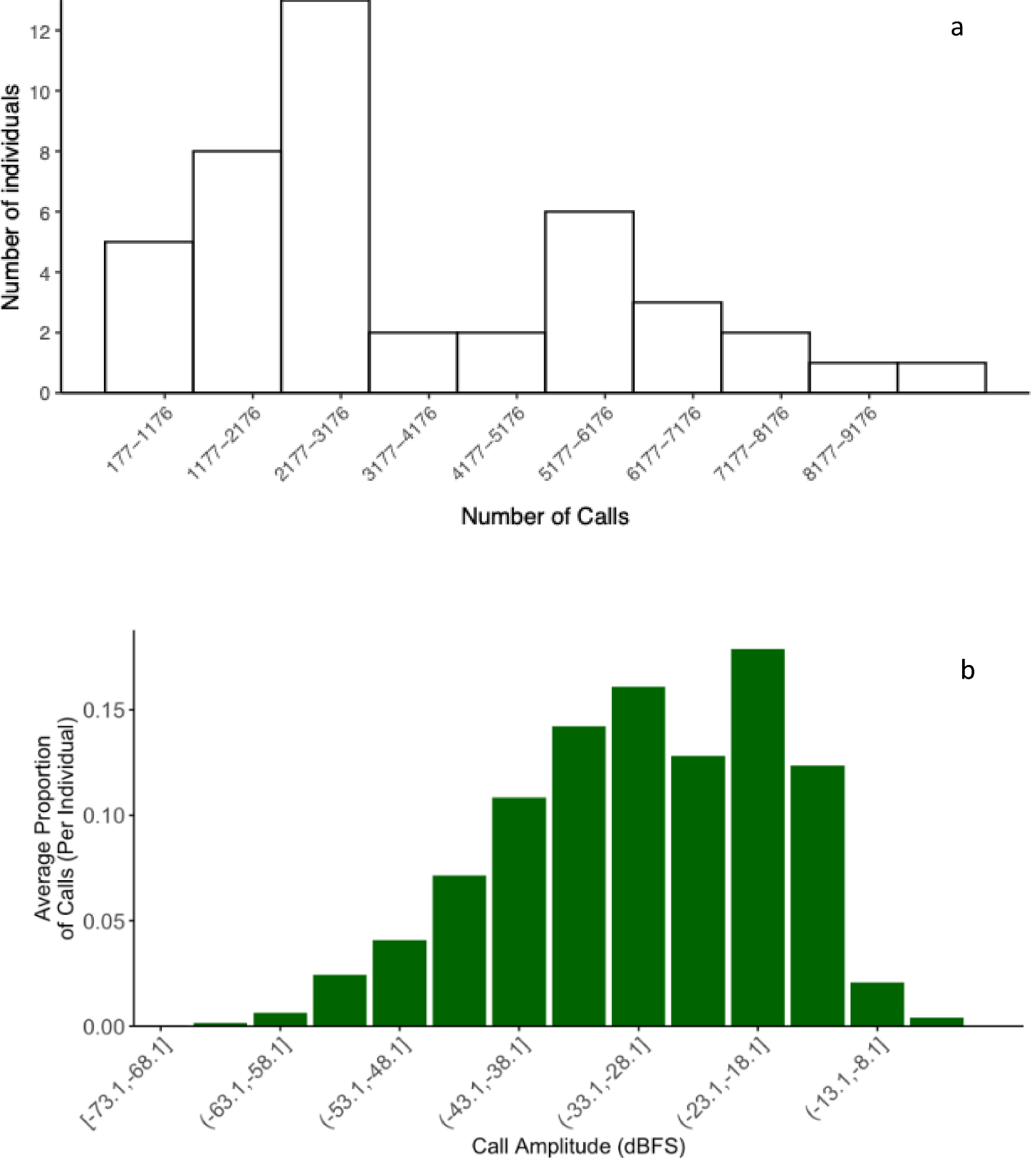
(**a**) Number of detected focal calls per adult individual; (**b**) Mean proportion of calls per individual along the gradient of amplitude (range − 74.0 to −2.5 dBFS)

**Fig. 5:**
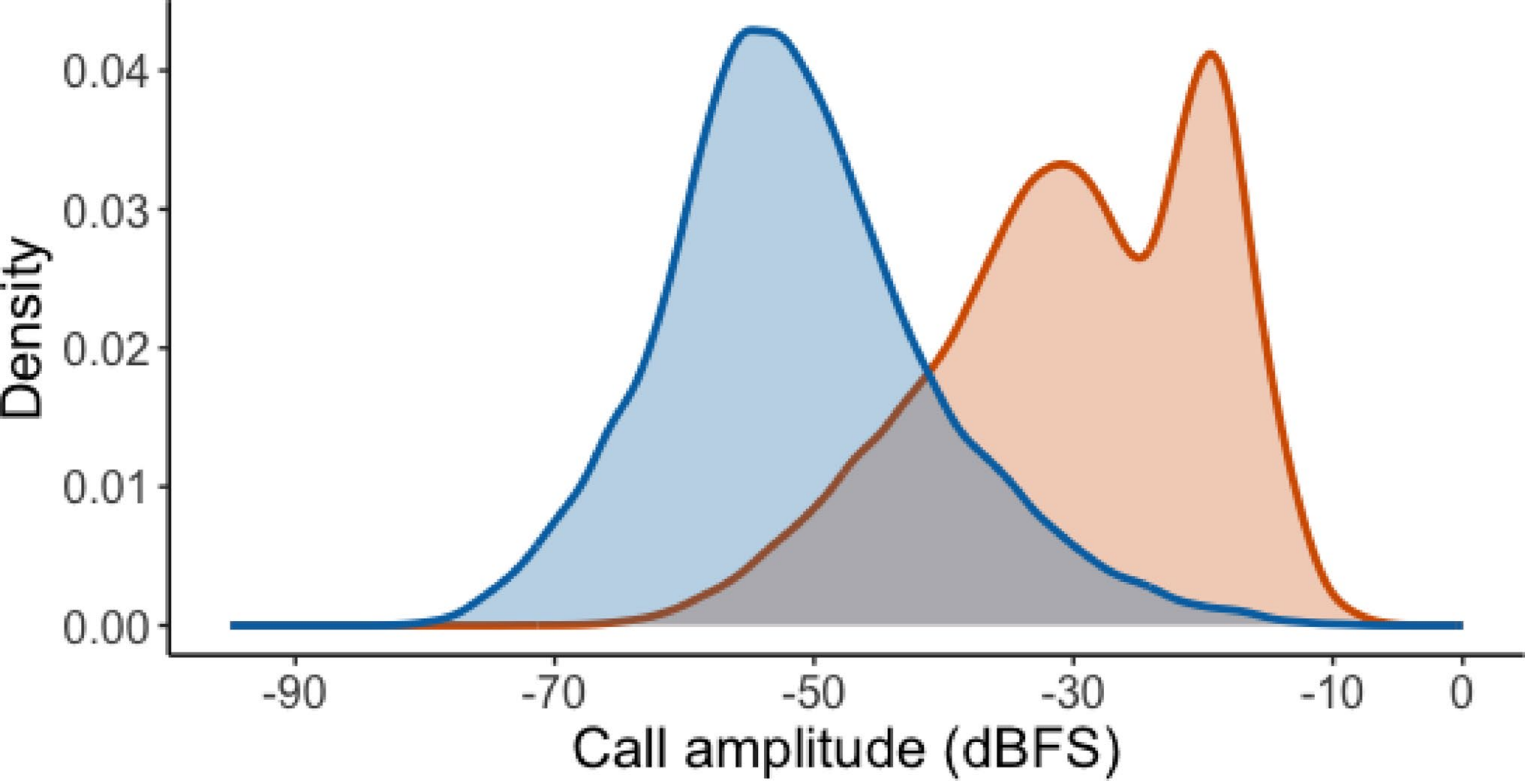
Smoothed density curves of call amplitudes (dBFS) for chick (blue) and adult (orange) crow vocalizations. Density represents the relative distribution of calls within each group, normalized to account for differences in sample size

The final test results of our trained model are presented in (Table 1). The model detected 127,750 adult vocalizations (mean = 2971 ± 356 number of calls per individual; range 177–8558; see Fig. 4a) across a wide amplitude range (from − 73.1 to −2.52 dBFS), enabling the recording of both subtle, soft calls and powerful, long-distance ‘kaa’ calls. The mean proportion of calls per individual varied along the amplitude gradient (Fig. 4b), with intermediate-amplitude calls being more frequent than either loud or very soft ones. The potential of our system to record non-focal, close-range calls is illustrated by our analysis of crow chick vocalizations, which are invariably directed at nearby individuals at the nest. Figure 5 shows that the amplitude range of chick calls overlaps with the lower end of adult crow call amplitudes, suggesting that our system can effectively capture even the quietest sounds, whether focal or not.

**Table 1:** Results of the trained detection model

	False negatives (FN)	False positives (FP)	True positives (TP)	Precision	Recall	F1
Macro- averaged	–	–	–	0.788522	0.773889	0.780108
Focal adult	378	293	1433	0.830243	0.791275	0.810291
Non-focal adult	251	182	867	0.826501	0.775491	0.800184
Crow chick	1834	1395	4818	0.775470	0.724293	0.749008
Cuckoo chick	591	937	2432	0.721875	0.804498	0.760951

### Signal-to-noise ratio

**Fig. 6:**
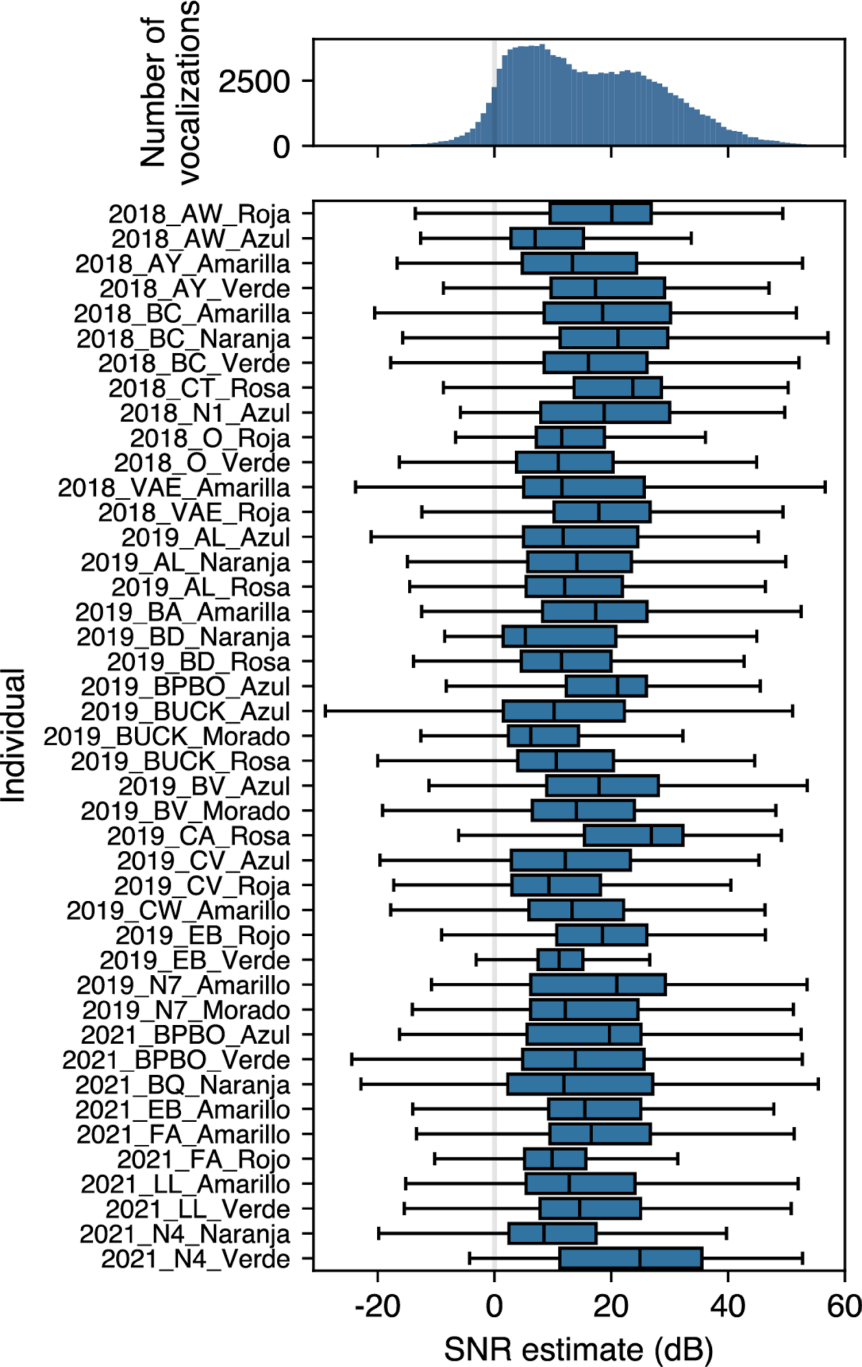
Top: histogram of estimated signal-to-noise ratio across all focal vocalizations from all individuals. The majority of vocalizations have signal-to-noise ratio above 0 dB (grey line). Bottom: distribution of signal-to-noise ratio per individual. Box shows the three quartile values, points more than 1.5 times the interquartile range from outer quartiles are not shown. All individuals show good median SNRs (> 5 dB), demonstrating that obtained vocalizations are suitable for subsequent analyses

The vocalizations of the focal individuals recorded by the tags could be intermingled with self-movement noise (e.g., flapping wings, landing, splashing), environmental noise (e.g., wind, vehicles) and sounds from other species. Good estimated SNRs were obtained for the detected focal vocalizations (Fig. 6). The range of median estimated SNR across individuals was 5.27 to 26.83 dB, with an average median of 14.68 dB. All individuals have lower quartiles above 1 dB, indicating that a high proportion of sounds (including low-amplitude calls) are suitable for subsequent analyses.

### Acceleration signatures of dives

**Fig. 7:**
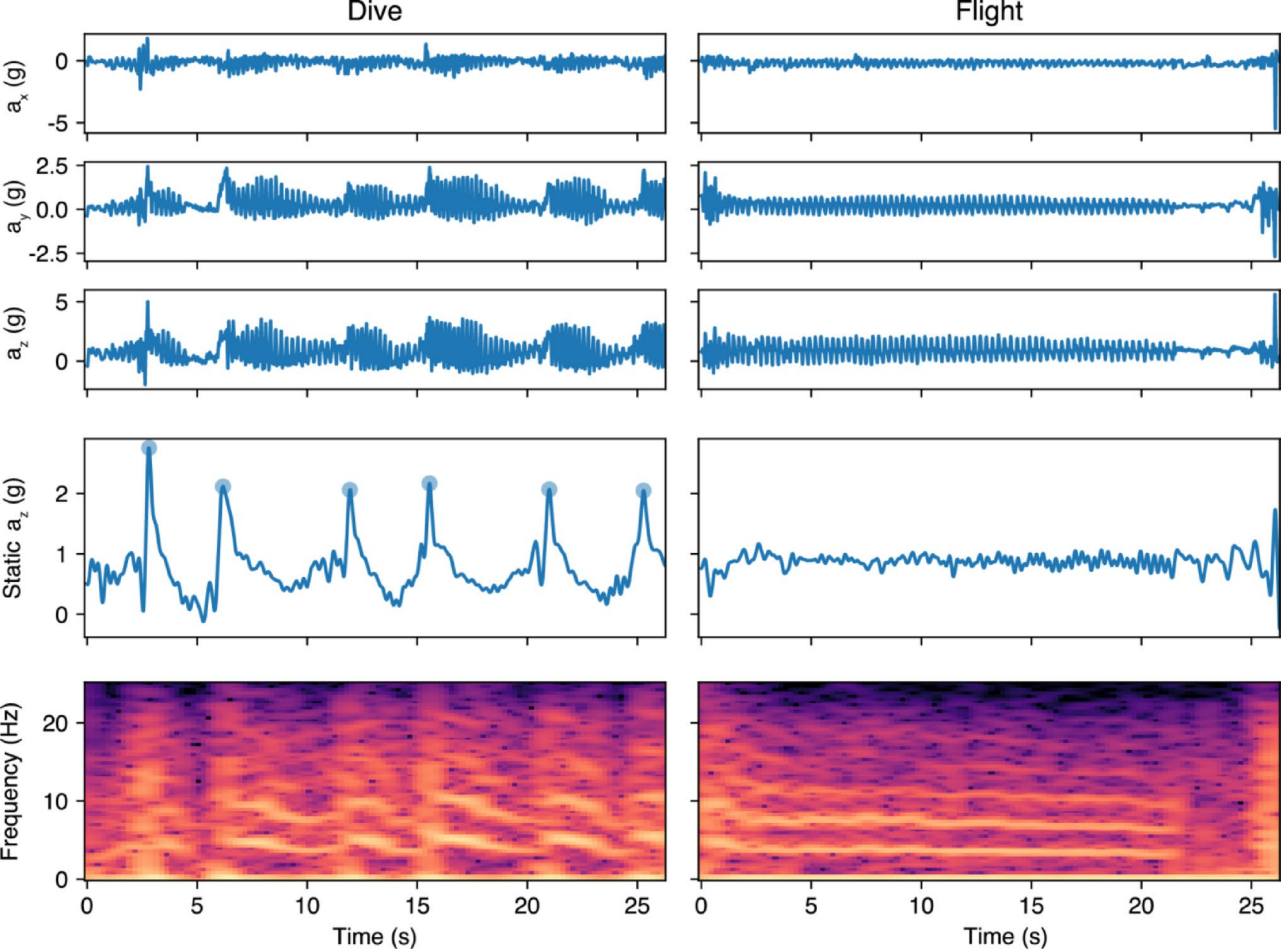
Left: portion of anti-predatory dive segment, right: flight from the nest. Top: raw triaxial acceleration. Middle: static acceleration in the z-direction, with dots indicating detected peaks. Bottom: spectrogram of raw acceleration in the z-direction, parameters given in Methods. Anti-predatory dive segment contains repeated segments showing peaks in the static acceleration and modulation in the dominant frequency. Flight from the nest shows a relatively steady dominant frequency (corresponding to wingbeat frequency) with an impulse at landing

Figure 7 shows two highly distinct examples of an anti-predatory flight compared to a flight from the nest, though overall both types of flights were variable. All segments are provided in the Supplementary Information (Fig. S1 and S2). We found that 11 of 20 dive segments contained at least one peak in the static acceleration in the z-direction, while 3 of 20 non-dive flight segments contained a peak (Fisher’s exact test, *p* = 0.018).

### MiniDTAG impact

**Fig. 8:**
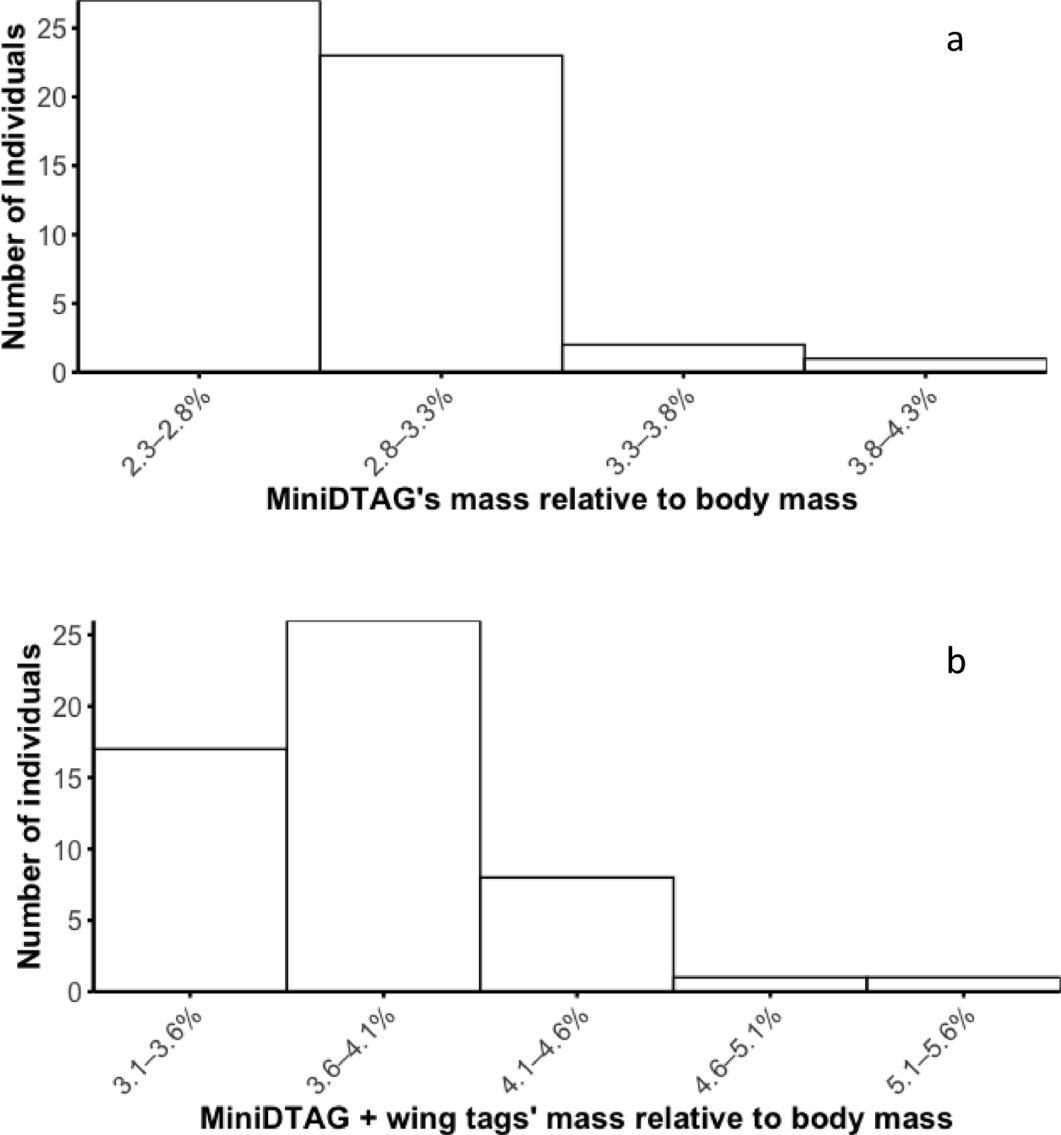
Percentage of crow body mass represented by the MiniDTAG alone (**a**), or in combination with the wing tags and ring (**b**)

Tagged crows weighed on average 475.20 ± 49.31 g (range 345–580 g) so the miniDTAG weight plus battery and transmitter accounted on average for the 2.80 ± 0.0004% of the crow body mass (range 2.28–3.83%; Fig. 8a). With the two wing tags and the coloured ring, the weight borne increased to 4.03 ± 0.0006% of body mass on average, with one tag exceeding the 5% body size threshold (5.51%; Fig. 8b).

**Fig. 9:**
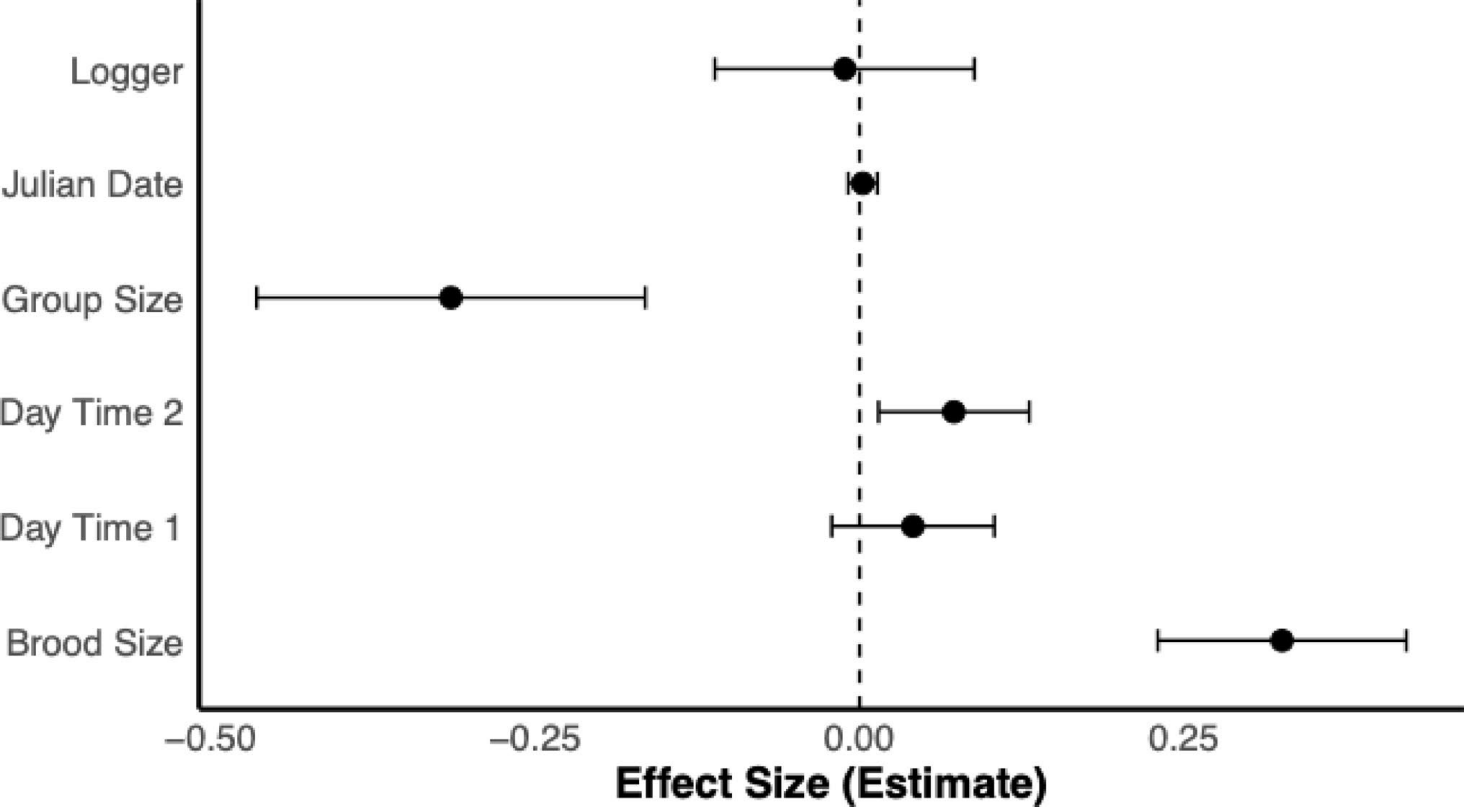
Forest plot showing the effect sizes (± 95% CI) of various predictors on brood provisioning behavior in the carrion crow. Effect sizes are derived from a Linear Mixed Model fitted by Restricted Maximum Likelihood (REML)

We found that group size, brood size and time of the day significantly influenced brood provisioning effort (95% Confidence Interval of effect size estimate not including zero; Fig. 9, Table S1), confirming previous results (Canestrari et al. 2005). Carrying a miniDTAG, instead, showed a very small negative effect, corresponding to a reduction of 0.61 feeds per hour in tagged birds.

**Fig. 10:**
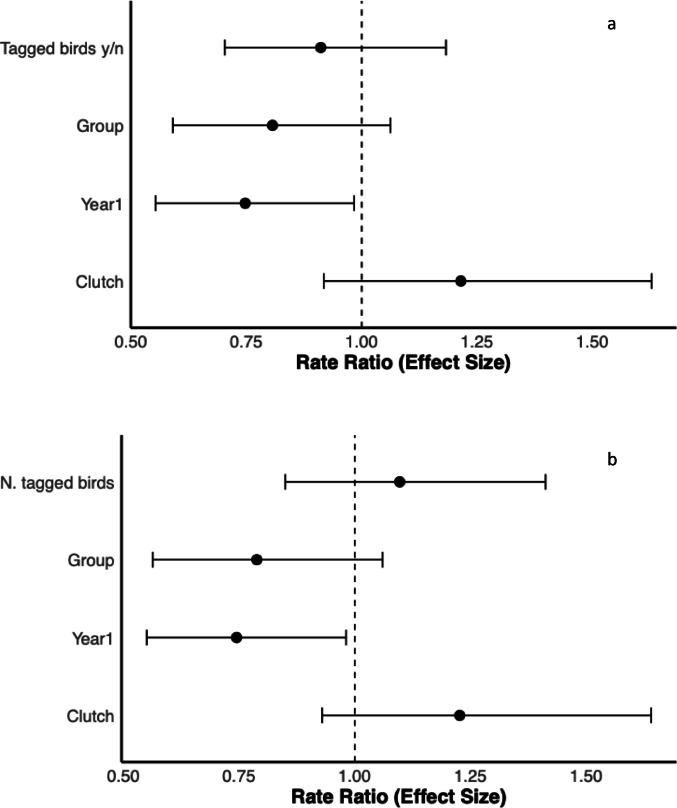
Forest plot illustrating the effect sizes (± 95% CI) of various predictors on the number of fledglings produced in carrion crow nests. Effect sizes were estimated using two General Linear Models (GLMs) with a Poisson distribution and a log link function: one assessing the effect of the presence or absence of tagged birds within the group (**a**), and the other considering the actual number of tagged individuals per group (**b**)

The presence of at least one tagged bird in the breeding group showed a minimal impact on the reproductive success, with a decrease of 0.13 fledglings as compared to groups of untagged crows (Fig. 10a, Table S2). Similarly, the actual number of tagged birds per group showed minimal influence, in this case with an increase of 0.14 fledglings per each additional tagged member (Fig. 10b, Table S3).

## Discussion

The MiniDTAG enabled the recording of high-quality crow vocalizations in their natural environment with unprecedented detail, capturing even low-amplitude calls that would have been beyond the reach of directional microphones. This is crucial for understanding the vocal communication system of this species. In this population, crows live in cohesive kin groups that are highly territorial and rapidly evict intraspecific intruders (Baglione et al. 2002, 2005). The observed prevalence of intermediate- to low-amplitude calls suggests that most vocalizations are directed toward members of the same group rather than individuals outside it. The MiniDTAG makes it possible to document this segment of the crow vocal repertoire, which, according to current theory (Leighton 2017), is expected to play a key role in coordinating cooperation among individuals engaged in communal tasks. In addition, our system proved effective in recording non-focal, close-range calls. This was exemplified by crow chick vocalizations—which are invariably directed at nearby individuals at the nest—being captured even at the lower end of the amplitude range. Recording both focal and non-focal close-range calls is essential for a more comprehensive understanding of crow communication and may be particularly valuable for inferring the function of different call types. As far as our data can show, the miniDTAG represents a meaningful step forward in this respect.

The tag retrieval rate was satisfactory, with only 13.5% of devices lost. The self-releasing attachment system proved effective, minimizing the time crows carried the tag while preventing premature detachment before the completion of most duty cycles. However, 66% of the tags did not complete their expected life cycle. While the average number of calls recorded per individual totaled nearly 3,000, reducing data loss remains a priority. Identifying the specific technical issues behind failures or suboptimal performance is challenging, but enhancing tag protection — such as with an epoxy coating — may improve durability. However, this could increase weight and reduce reusability. Future studies should also investigate whether alternative battery brands or models can enhance tag reliability.

We found that we were able to automate a large audio annotation task – detecting and classifying vocalizations – using machine learning techniques developed for bioacoustics. While we invested a substantial annotation effort (187.7 h of audio), this was only 4.3% of the total data. The detection model had high precision and recall, especially for adult crow vocalizations, making the detections reliable for answering future scientific questions. Since multiple crows can vocalize at one time, our model’s ability to isolate individual calls opens the door for a number of interesting future analyses. For example, periods with high calling activity, measured by the call rate in a period of time, can be identified based on our model’s detections. These high-activity periods can, in turn, be correlated with other behavioral measures, such as nest visits, while call rates can be compared across individuals from different social categories (breeders vs. helpers) or between sexes. We also found that a majority of detected vocalizations had good estimated signal-to-noise ratios, including across all focal individuals. A second application of the detected calls is to build a corpus of isolated crow vocalizations, to enable comparisons of vocal repertoire across individuals and groups. A similar effort has been undertaken in rooks (Martin et al. 2024), and there is potential for future comparative studies as more corvid repertoires are collected.

Future work could further improve the quality of the vocalization dataset. Since our annotations of caller identity were based on aural assessment of multiple distance cues, we expect mistakes to occur when these cues are weaker, as when two birds are nearby. A promising future approach would be to use level comparisons across multiple synchronized biologgers to assign the caller identity *(*Zeh et al. 2024). Also, denoising vocalizations could increase the signal-to-noise ratios (Sainburg et al. 2020).

### Accelerometry

Accelerometers are widely used in animal behaviour studies because they provide high-resolution, continuous data on activity patterns, energy expenditure, and specific behaviours in both wild and captive settings (Brown et al. 2013; Wassmer et al. 2020). A previous study (Hoffman et al. 2024) demonstrated the utility of accelerometers in inferring behavioural states in carrion crows, particularly in the context of parental care. In the present study, we investigated whether accelerometer data could also potentially be used to reveal natural encounters between crows and predators, events that are notoriously difficult to observe in the wild. These encounters are of particular interest, as they often elicit communal mobbing behaviour, which, alongside brood provisioning, is a key task performed cooperatively by crow group members.

We found that anti-predatory dive segments differed from other flight segments in one hand-designed feature, the presence of peaks in the z-direction of static acceleration. While the two categories were not discriminated perfectly, this choice of categories was conservative: for flights without anti-predatory dives, we chose flights when birds left the nest, which are likely more similar to anti-predatory dives than other flights due to downward movement from the nest. This result provides a proof-of-concept that the accelerometer data may aid in characterizing anti-predatory behavior, and could be extended in future work by more extensive feature design. For example, discrimination may be improved by incorporating behavior durations or modulation in the dominant frequency. The dataset did not contain sufficient examples to use machine learning classifiers, but future work could increase annotation effort to take advantage of these methods (Hoffman et al. 2024). The addition of audio features, such as a high density of non-focal signals due to nearby signallers or the presence of a particular vocalization type, may further serve to identify and characterize cooperative mobbing.

Another potential application of accelerometry is linking the vocalizations of focal or bystanding individuals, as recorded by the microphone, with the subsequent motion responses of tagged crows. Identifying such patterns could provide insights into the type of information different calls convey. Additionally, multichannel data could help explore the motion and acoustic responses of focal birds to interspecific calls (e.g., from a predator) or environmental noises. Finally, motion sensors could be used to infer body postures associated with specific call types (Shepard 2008), improving our understanding of sound production mechanisms in crows and the possible multimodal nature of their communication repertoire (Higham and Hebets 2013).

### Impact of the logger

A phylogenetically controlled meta-analysis by Bodey et al. (2017) suggests that, in birds, biologging significantly affects parental care and reproductive success. In our study, we assessed the impact of the miniDTAG on these variables using a relatively large sample of individuals and nests. Our video-recorded observations at the nests enabled precise quantification of the birds’ provisioning efforts by counting the exact number of feeds delivered to the brood during each feeding event. Previous research on this cooperative carrion crow population has shown that brood provisioning is highly demanding, leading to substantial body weight loss, which increases linearly with the effort invested in feeding (Canestrari et al. 2007). As a result, crows finely adjust the amount of food carried to the nest to balance chick requirements with their own energy needs, prioritizing self-maintenance under worsening conditions (Canestrari et al. 2004, 2010). Notably, crows whose wing loading was temporarily increased by 11.3% responded by significantly reducing their feeding rate — by an average of 29.3% — and, in some cases, ceased provisioning entirely (Baglione et al. 2010).

In contrast, the effect of carrying the data logger was much more limited, causing an average reduction of fewer than one feed per hour (considering that a crow can deliver up to 25 feeds per hour). The impact on reproductive success was also minimal. Whether considering the presence or absence of tagged crows in a group, or the actual number of tagged individuals, the change in fledgling number remained consistently close to zero (–0.13 and + 0.14, respectively). Compared to the average reproductive output of non-predated broods in this population (2.39 ± 0.05 fledglings), these differences represent less than 6% of the mean. Such deviations fall well within the normal range of variation (SD = 1.04 fledglings) expected from ecological or individual factors and are unlikely to influence population dynamics or long-term fitness.

Quantifying and minimizing biologgers’ risks across taxa remains a topic of debate, primarily due to the lack of standardized reporting in most studies. The failure to report the effect size of logger deployment on the studied variables is a major issue that hinders reliable assessments of tag impact. Even the most fundamental information—such as the number of deployments and individuals tagged, the mean mass of the studied animals, the mass of the deployed device, methods of attachment, and the total duration of tag deployment—is omitted in approximately two-thirds of published studies (Bodey et al. 2017). As biologging technology is likely to expand rapidly in the field of avian bioacoustics, we encourage researchers to measure and report the impacts of tags, as doing so will help refine best practices that safeguard both animal welfare and data reliability.

## Concluding remarks

Biologging is opening new frontiers for the study of communication in birds. The design, weight, and attachment method of the miniDTAG appear to minimize its impact on carrion crows while allowing for the collection of high-quality acoustic and kinetic data. Optimizing the tags´ operating schedule to coincide with peak vocal activity could reduce battery weight, making it more suitable for use in smaller corvid species. In addition, using thinner balloons for attachment may shorten the duration that a bird carries the tag but also increases the risk of premature detachment and subsequent data loss.

Minimizing the number of instrumented birds is an important ethical concern. In this respect, conducting pilot tests to estimate tag failure and retrieval rates in the intended deployment environment, as well as the degradation rate of the rubber used to secure the tag, will enable researchers to refine the study design and ensure a balance between data collection and the number of animals handled. These tests would be also valuable to improve tag design, with the aim of ensuring a more reliable data collection.

Furthermore, artificial intelligence plays a key role in automating data processing, particularly in detecting calls within large audio datasets, significantly enhancing the efficiency of biologging studies. Currently, this task requires a level of expertise that most biologists do not possess. Recent efforts have focused on developing user-friendly applications that can be operated by non-specialists in AI (Hoffman et al. 2025; Robinson et al. 2024). Replacing custom AI data processing pipelines with such tools in future studies would represent a significant advancement in the field.

## Supplementary Information

Below is the link to the electronic supplementary material.

Supplementary Material 1

## Data Availability

Data available at: https://figshare.com/s/4e7598244ec1ba63aaed.
