# Supplementary material for: Capturing vocal communication in a free-living corvid: high-resolution data from low-impact miniaturized tags
Source: Anim Cogn. 2025 Oct 29;28(1):85. doi: 10.1007/s10071-025-02018-0 (PMC12572106; doi:10.1007/s10071-025-02018-0)
Supplement: Supplementary file 1 — Supplementary Material 1 [file 10071_2025_2018_MOESM1_ESM.docx]

SUPPLEMENTARY MATERIAL

Table S1. Variables associated to chick provisioning behaviour in the carrion crows. Results of Linear Mixed Model fit by Restricted Maximum Likelihood.

| Term | Estimate | Std. Error | CI Lower | CI Upper | Df | t-value | p-value |
| --- | --- | --- | --- | --- | --- | --- | --- |
| Intercept | 1.06 | 0.53 | 0.01 | 2.11 | 69.98 | 2.01 | 0.048 |
| Group size | -0.32 | 0.08 | -0.47 | -0.17 | 49.70 | -4.23 | << 0.01 |
| Julian date | 0.01 | 0.01 | -0.01 | 0.014 | 76.69 | 0.43 | 0.67 |
| Brood size | 0.33 | 0.05 | 0.23 | 0.42 | 106.08 | 6.74 | << 0.01 |
| Social category (Breeder) | 0.15 | 0.08 | -0.02 | 0.32 | 50.51 | 1.82 | 0.07 |
| Day time  5:00 – 10:00 | 0.04 | 0.03 | -0.02 | 0.10 | 525.07 | 1.29 | 0.20 |
| Daytime  10:01 -15:00 | 0.07 | 0.03 | 0.02 | 0.13 | 524.08 | 2.46 | 0.01 |
| Logger (yes) | -0.01 | 0.05 | -0.11 | 0.09 | 257.29 | -0.23 | 0.82 |

Table S2. Variables associated with reproductive success in the carrion crows, including a dichotomic term that indicates presence/absence of individuals tagged with the miniDTAG in the social group. Results of General Linear Model.

| Term | Estimate | Std. Error | CI Lower | CI Upper | z-value | p-value |
| --- | --- | --- | --- | --- | --- | --- |
| Intercept | -0.09 | 0.94 | -1.78 | 1.53 | -0.11 | 0.91 |
| Tagged (yes) | -0.09 | 0.13 | -0.35 | 0.17 | -0.70 | 0.48 |
| Group size | -0.22 | 0.15 | -0.53 | 0.06 | -1.44 | 0.15 |
| Year 2018 | -0.29 | 0.15 | -0.59 | -0.02 | -2.00 | 0.046 |
| Clutch size | 0.20 | 0.15 | -0.09 | 0.49 | 1.33 | 0.18 |

Table S3. Variables associated with reproductive success in the carrion crows, including the number of individuals tagged with the miniDTAG in the social group. Results of General Linear Model.

| Term | Estimate | Std. Error | CI Lower | CI Upper | t-value | p-value |
| --- | --- | --- | --- | --- | --- | --- |
| Intercept | -0.17 | 0.83 | -1.83 | 1.42 | -0.21 | 0.84 |
| Tagged (yes) | 0.09 | 0.13 | -0.16 | 0.35 | 0.72 | 0.47 |
| Group size | -0.24 | 0.16 | -0.57 | 0.06 | -1.48 | 0.14 |
| Year 2018 | -0.30 | 0.15 | -0.60 | -0.02 | -2.01 | 0.04 |
| Clutch size | 0.20 | 0.15 | -0.07 | 0.50 | 1.41 | 0.16 |


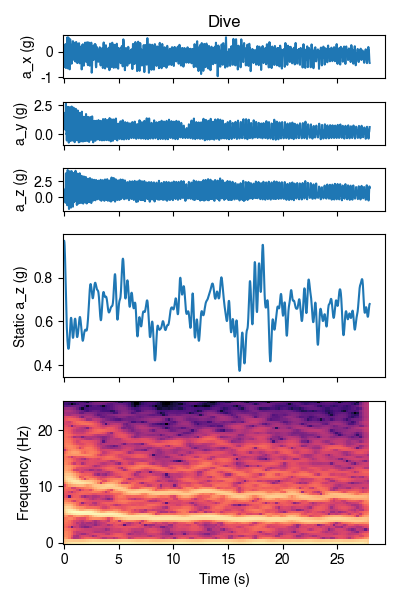

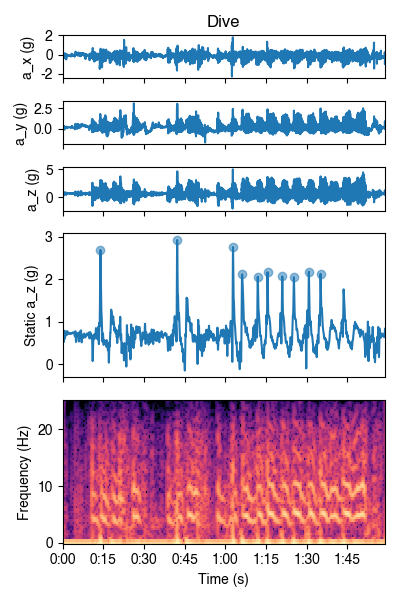

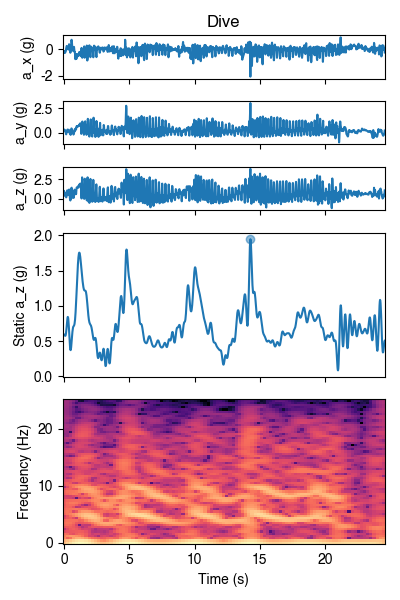

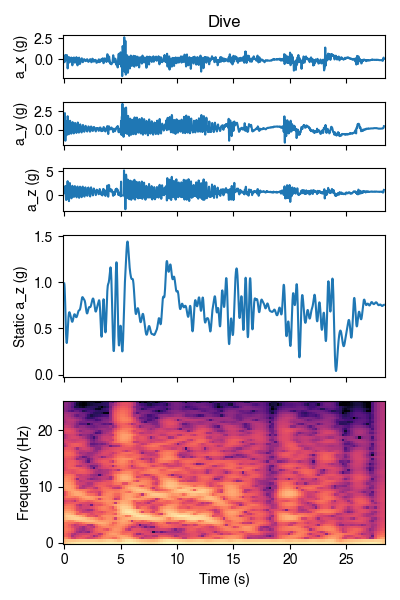

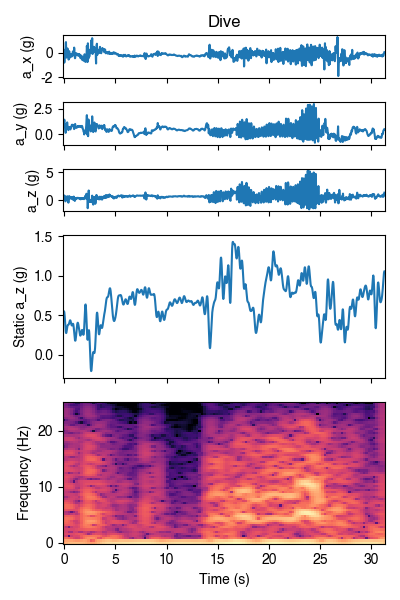

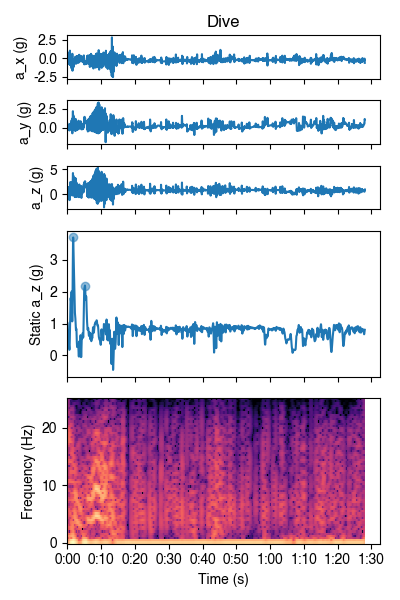

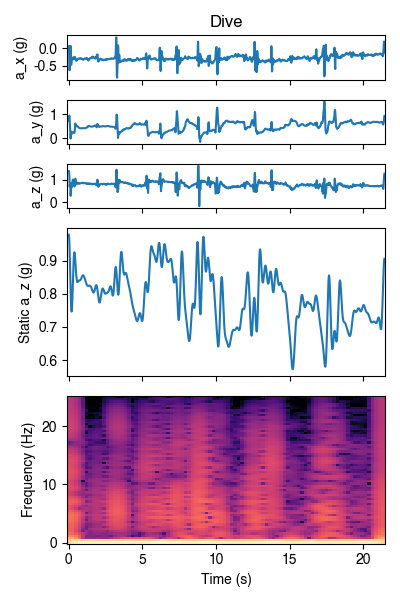

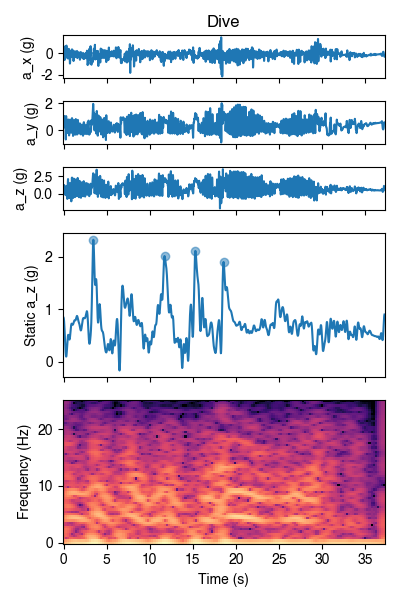

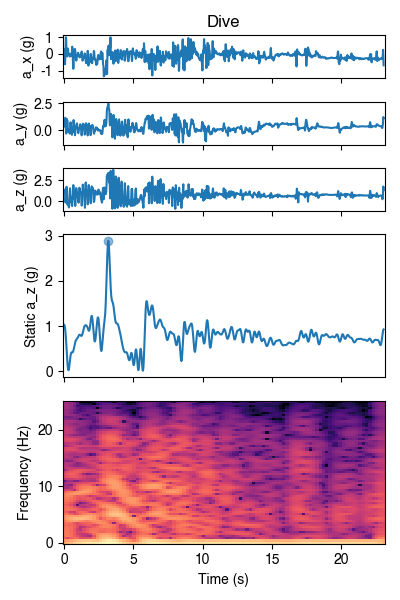

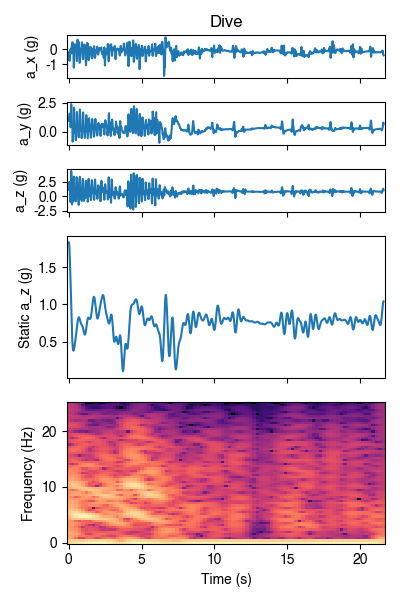

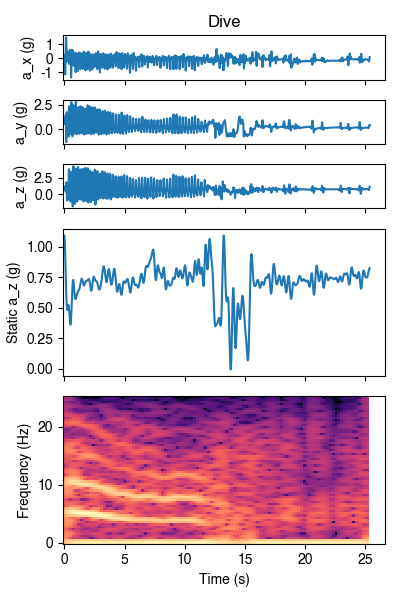

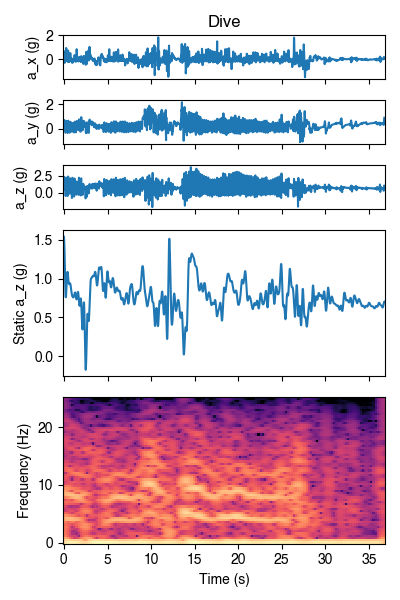


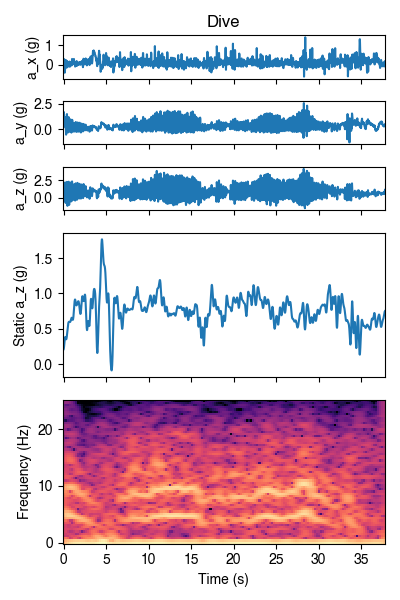

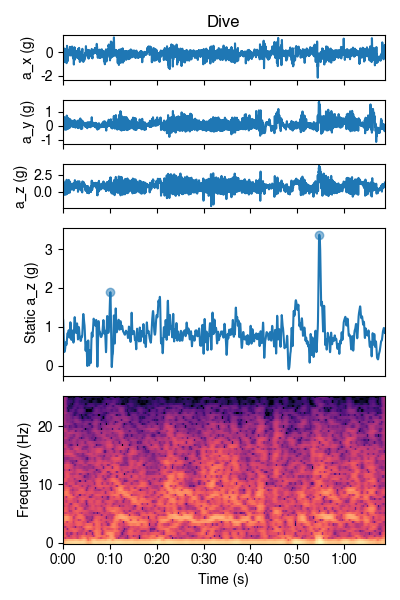

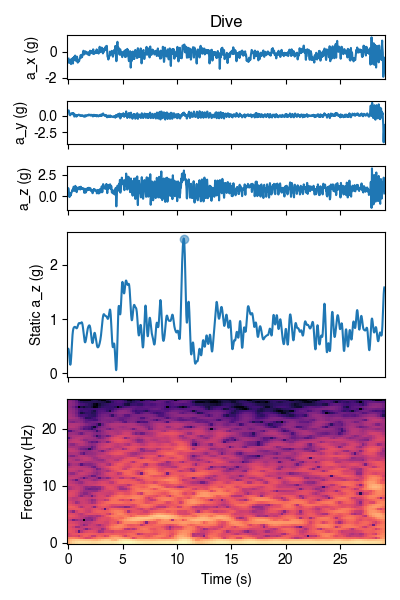

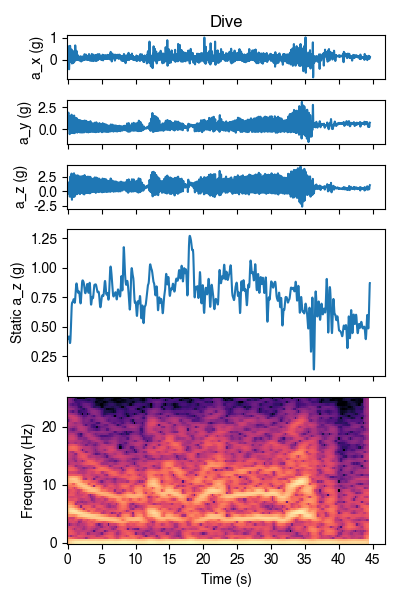

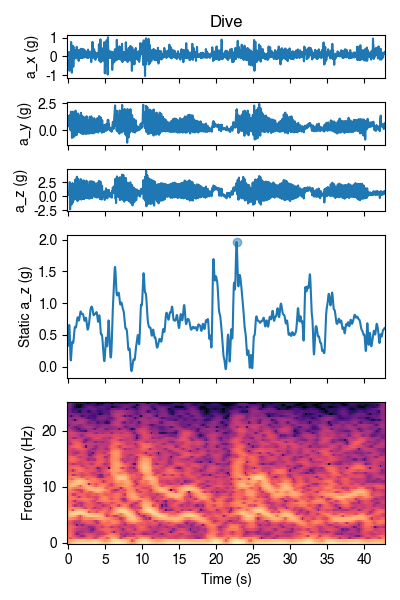

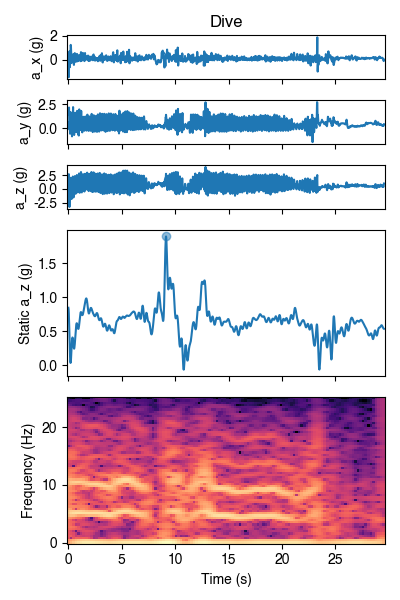

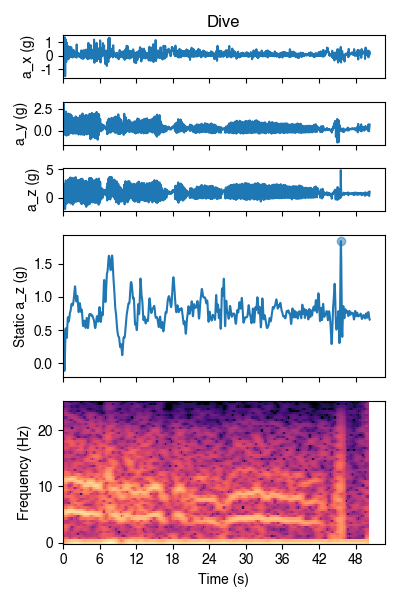

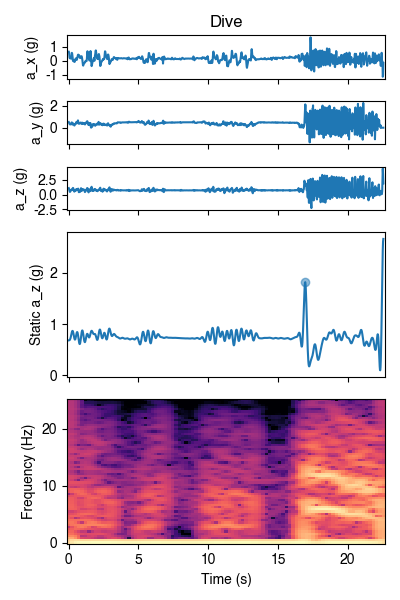


Fig. S1. Portions of anti-predatory dive segments. Top: raw acceleration in the z-direction. Middle: static acceleration in the z-direction, with dots indicating detected peaks. Bottom: spectrogram of raw acceleration in the z-direction, parameters given in Methods.


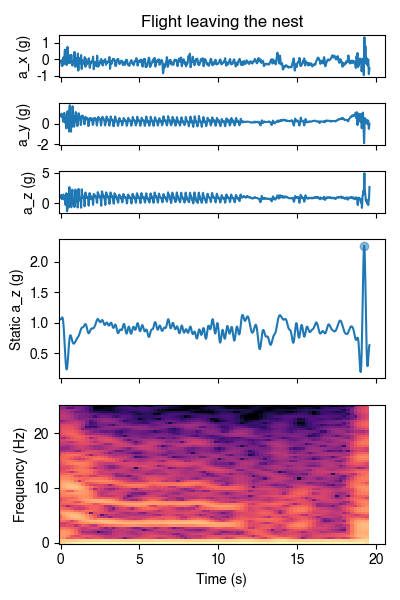

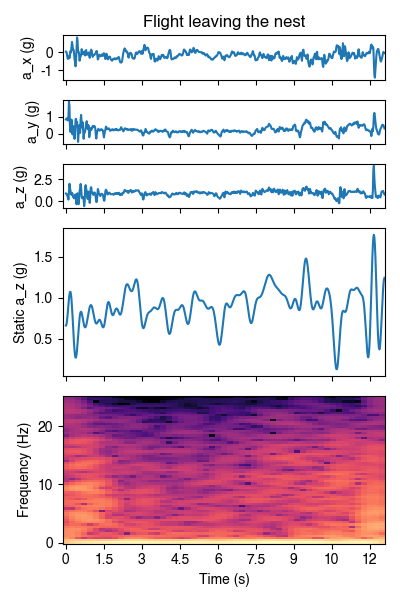

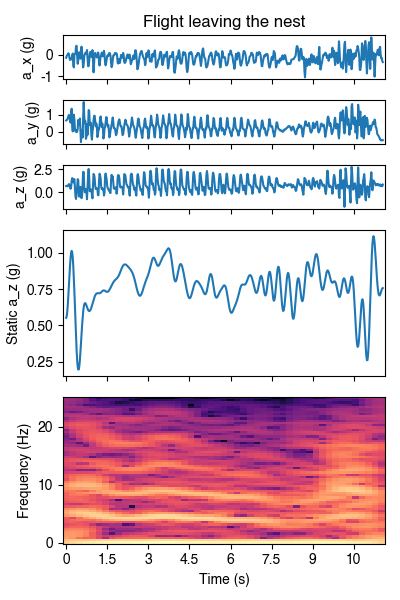

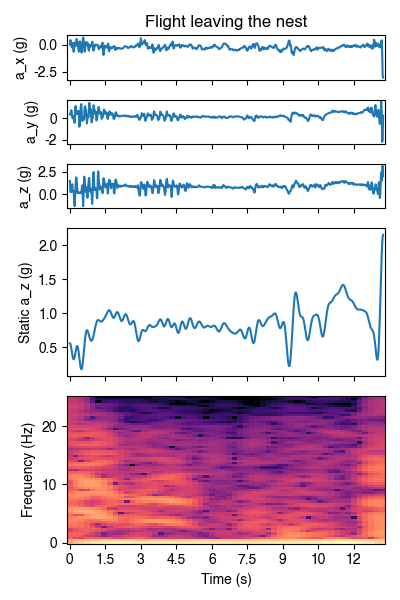

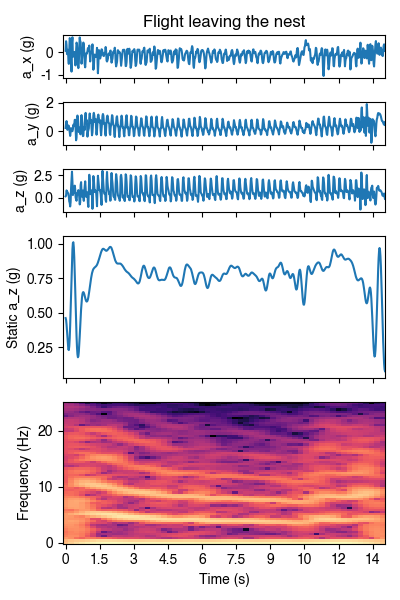

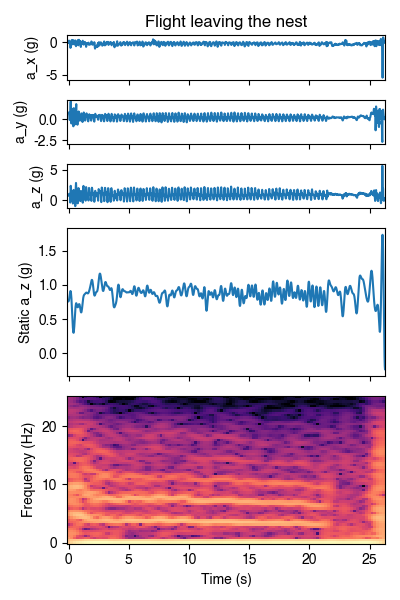

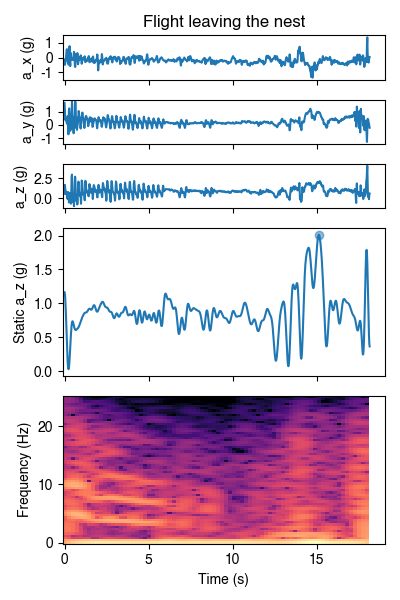

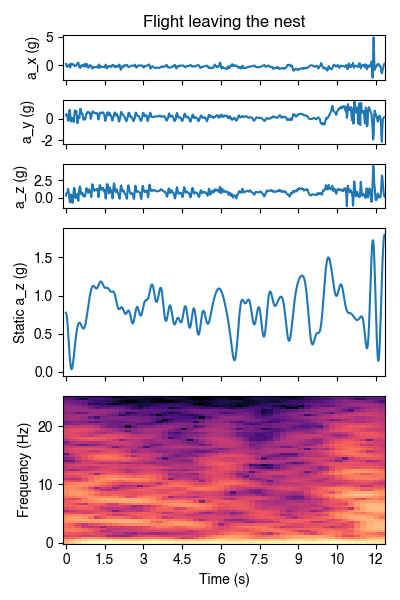

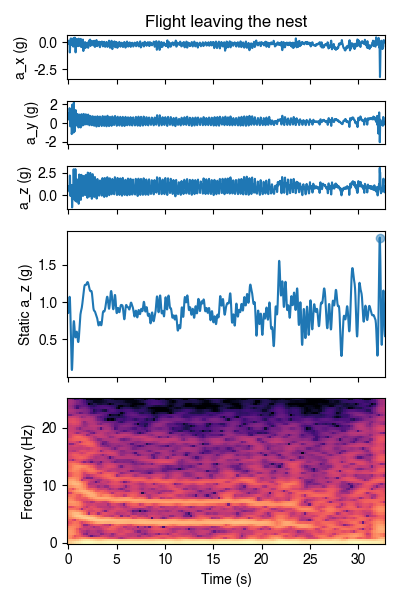

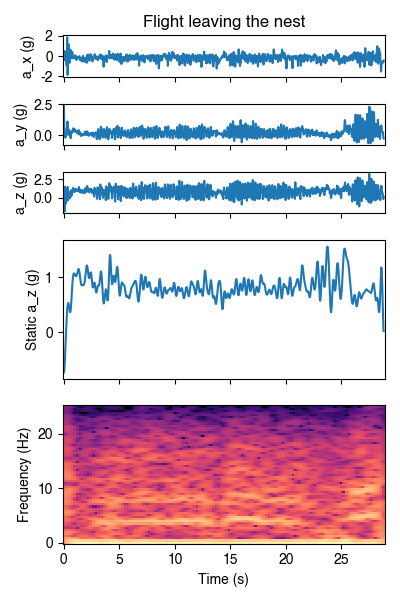

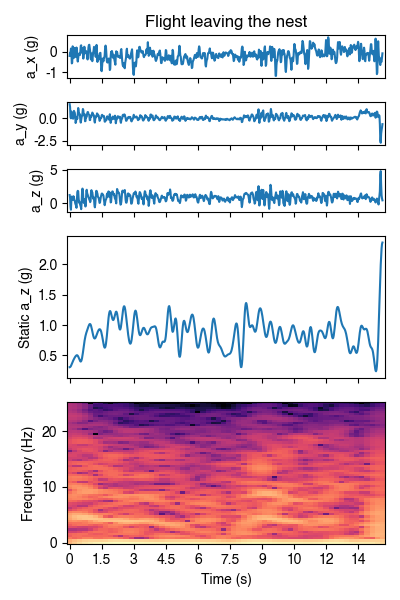

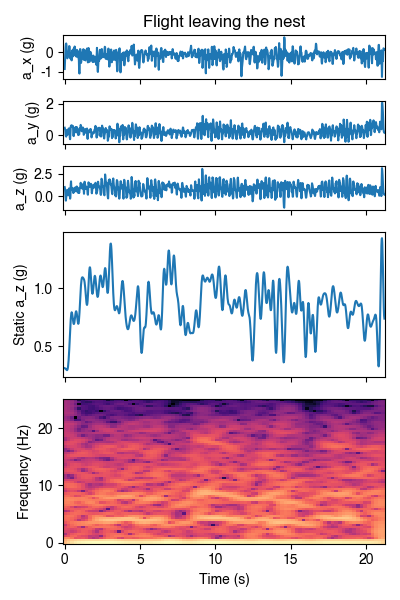

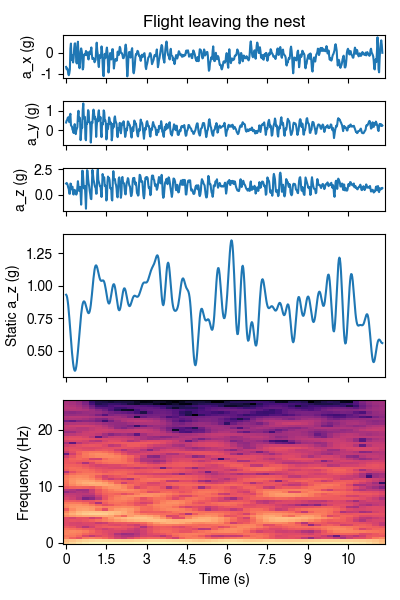

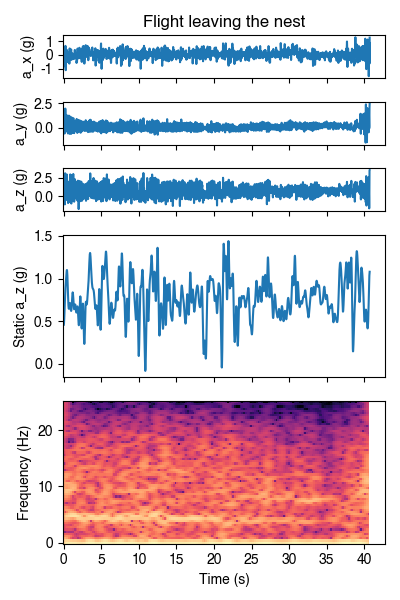

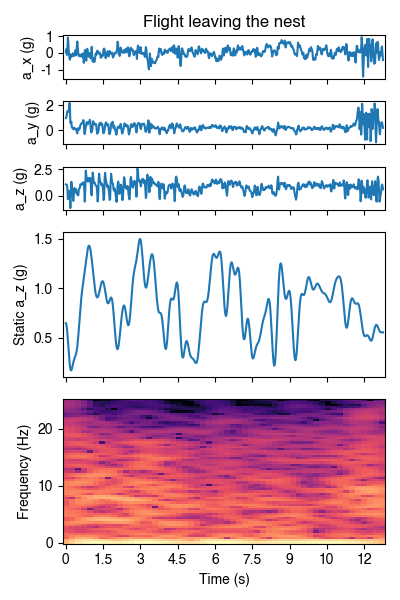

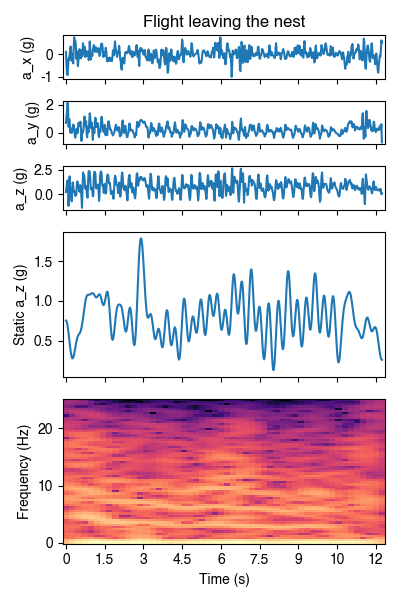

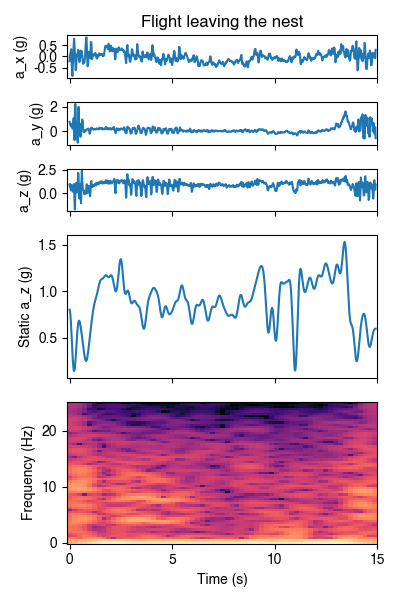

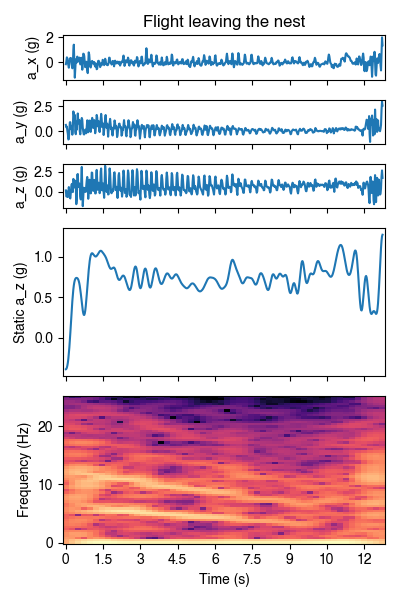

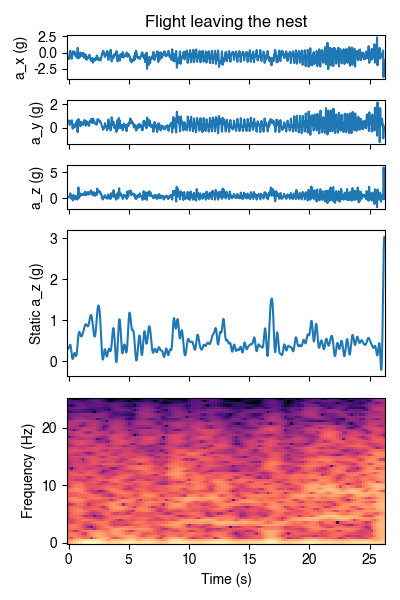

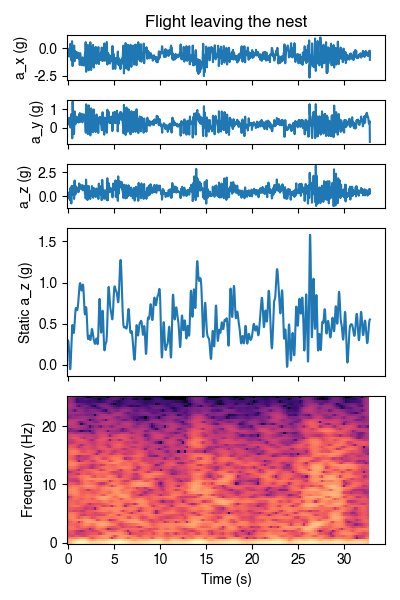


Fig. S2. Portions of segments of flights during nest departure. Top: raw acceleration in the z-direction. Middle: static acceleration in the z-direction, with dots indicating detected peaks. Bottom: spectrogram of raw acceleration in the z-direction, parameters given in Methods.
